# Aluminum and Fluoride Stresses Altered Organic Acid and Secondary Metabolism in Tea (*Camellia sinensis*) Plants: Influences on Plant Tolerance, Tea Quality and Safety

**DOI:** 10.3390/ijms24054640

**Published:** 2023-02-27

**Authors:** Anqi Peng, Keke Yu, Shuwei Yu, Yingying Li, Hao Zuo, Ping Li, Juan Li, Jianan Huang, Zhonghua Liu, Jian Zhao

**Affiliations:** 1 Key Laboratory of Tea Science of Ministry of Education, College of Horticulture, Hunan Agricultural University, Changsha 410011, China; 2 State Key Laboratory of Tea Plant Biology and Utilization, Anhui Agricultural University, Hefei 230036, China

**Keywords:** aluminum and fluoride, organic acid, secondary metabolite, tea quality, tea safety

## Abstract

Tea plants have adapted to grow in tropical acidic soils containing high concentrations of aluminum (Al) and fluoride (F) (as Al/F hyperaccumulators) and use secret organic acids (OAs) to acidify the rhizosphere for acquiring phosphorous and element nutrients. The self-enhanced rhizosphere acidification under Al/F stress and acid rain also render tea plants prone to accumulate more heavy metals and F, which raises significant food safety and health concerns. However, the mechanism behind this is not fully understood. Here, we report that tea plants responded to Al and F stresses by synthesizing and secreting OAs and altering profiles of amino acids, catechins, and caffeine in their roots. These organic compounds could form tea-plant mechanisms to tolerate lower pH and higher Al and F concentrations. Furthermore, high concentrations of Al and F stresses negatively affected the accumulation of tea secondary metabolites in young leaves, and thereby tea nutrient value. The young leaves of tea seedlings under Al and F stresses also tended to increase Al and F accumulation in young leaves but lower essential tea secondary metabolites, which challenged tea quality and safety. Comparisons of transcriptome data combined with metabolite profiling revealed that the corresponding metabolic gene expression supported and explained the metabolism changes in tea roots and young leaves via stresses from high concentrations of Al and F. The study provides new insight into Al- and F-stressed tea plants with regard to responsive metabolism changes and tolerance strategy establishment in tea plants and the impacts of Al/F stresses on metabolite compositions in young leaves used for making teas, which could influence tea nutritional value and food safety.

## 1. Introduction

Tea is one of the most consumed non-alcoholic beverages and has become increasingly popular because of its rich tastes and potential health benefits, which are largely attributable to the high levels of tea-characteristic secondary metabolites, including catechins, caffeine, and theanine [1,2]. Tea plants are also a typical aluminum ion (Al in short) and fluorine anion (F in short) hyperaccumulator because tea plants used to grow in acidic soils containing high concentrations of Al and F in the tropical and subtropical regions of mountainous areas [3,4,5,6]. The average content of F in the soils of tea plantations in China is 540, ranging between 190 and 1100 mg/kg in most areas, but in southwest provinces with F mines or polluted soils, such as GuiZhou, the soil F content can be over 2000 mg/kg [7,8,9,10]. F is easily dissolved in acidic water and readily taken up by tea plant roots, and F concentrations in soils are highly correlated with F levels in tea leaves [7,8,9]. Tea plant leaves can accumulate up to 20,000 mg Al/kg and 1000–3000 mg F/kg in mature and old leaves and more than 600 mg Al/kg, 100–300 mg F/kg in young leaves, which are hundreds of times higher than in other crops [11]. Over-uptake of these elements by drinking teas, particularly teas made from old leaves and stems such as brick tea, can cause serious health problems, such as permanent damage to key enzymes and the circulatory, renal, brain, bone, and central neuron systems [5,12,13,14]. However, soils in many regions where large acreages of tea plants are planted are very acidic, contain high levels of F and Al, and are contaminated with carcinogenic heavy metals [8,9,12,15]. As one of the major factors that cause Al and F rhizotoxicities, the low pH values of tea plantation soils could be reduced continuously due to NH_4_ fertilization, proton and acid secretion by tea roots, and more frequently occurring acid rain under global climate changes [16,17,18]. Moreover, the accumulation of high levels of Al and F in tea leaves transported from tea roots also has been shown to negatively affect plant growth and development [19,20,21,22]. Excessive F causes both cell wall dysfunctions and intracellular damages [20,21,23]. Excessive Al entering into the plant cells destroys plant cell membrane, chloroplast structures, and enzyme activity, and causes metabolic process distortion, eventually resulting in early leaf loss or plant death [19].The biosynthesis and accumulation of major tea secondary metabolites, such as catechins and theanine, could be inhibited by Al and F stresses, which thereby reduces tea nutritional quality [12,23,24]. Therefore, there is a great need to reduce Al/F and other heavy metal accumulation in tea leaves through the genetic improvement of tea plant cultivars. However, so far, the understanding of the tea root uptake, vascular translocation, and leaf accumulation of Al/F and these toxic heavy metals are very limited, which largely prevents us from looking for efficient solutions for the breeding of safer tea plants [23].

Under Al stress, plant roots can secret organic acids to form Al-organic acid complexes, but it is not known whether F stress induces the exudation of organic acids to the tea root rhizosphere [3,4,25]. The Al can form different conjugates in tea plant tissues, such as Al-F in old leaves (AlF_2_^+^, AlF_3_^0^ and AlF_4_^−^ exist together) [26], Al-oxalate in root tips and saps, Al-citrate in shoot xylem saps, or Al-catechins in roots, which are considered as mechanisms of Al/F-hyperaccumulation and tolerance in tea plants [3,4,25,27,28]. Al and F stresses significantly modulated flavonoids, theanine, caffeine, and other amino acids in tea roots and leaves, which may contribute to Al and F detoxification on site [23,29,30,31,32]. Theanine is primarily synthesized in tea roots and could be translocated to the shoot tips, similar to Al and F translocation from tea roots to shoot tips [23,33]. While organic acids were widely considered as Al/F tolerant mechanism in many crops, the tricarboxylic acid cycle (TAC) and its metabolism in tea roots is not reported [23,34,35] in terms of enhancing plant tolerance to Al/F stresses and facilitating the transport and accumulation of Al/F and heavy metals to shoots, thereby impacting the food safety and nutrition quality of tea [14,20,36]. Thus, it is of particular interest to understand them for many purposes.

In this study, we studied organic acid biosynthesis in roots, their secretion into the root rhizosphere, and their possible connections to catechins, theanine, and caffeine biosynthesis pathways in both tea roots and shoots under Al and F stresses. By integrating transcriptome data and the relevant metabolite profiling of tea roots and leaves under Al and F stresses, we were able to look into their intriguing connections to consider tea nutritional quality and safety. The study shed new light on the root TAC, amino acid, and secondary metabolism in tea plant response Al/F stresses, tolerance strategies, and their potential effects on tea safety and nutritional quality.

## 2. Results

### 2.1. Analysis of Organic Acid Synthesis and Secretion in Tea Roots Treated with Al and F

**Figure 1:**
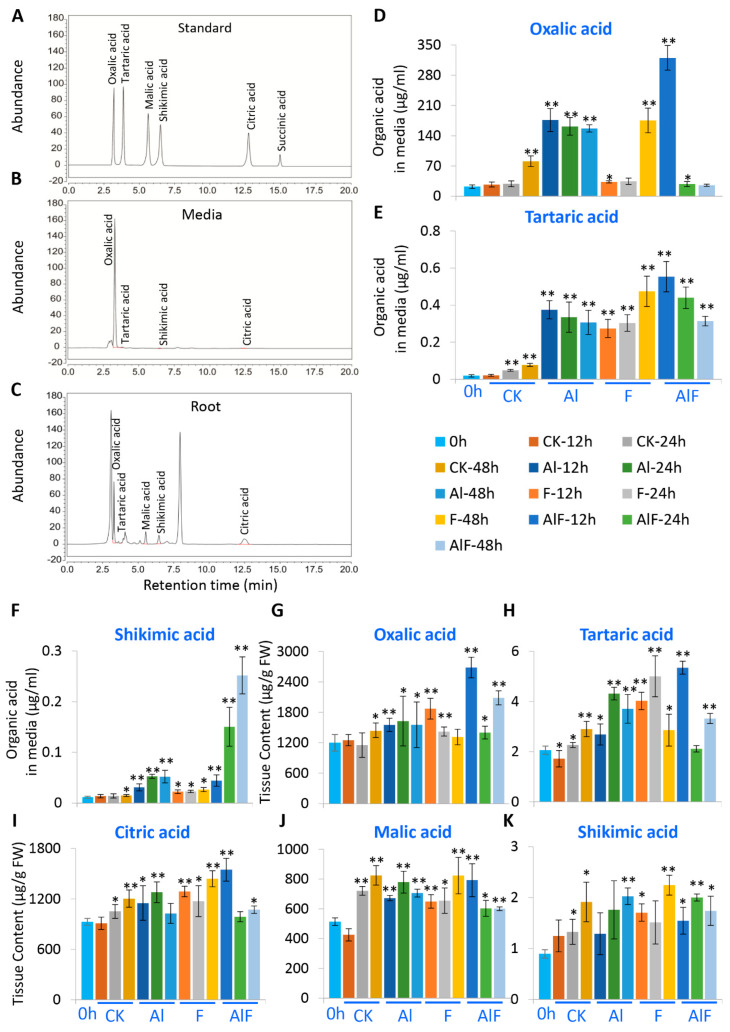
Al and F treatments promoted organic acid accumulation in tea roots and secretion into media. (**A**) The HPLC trace for organic acid standards. (**B**) Detections of oxalate, tartaric acid, citric acid, and succinic acid in tea root exudates. (**C**) Detections of organic acids in root tissues. (**D**) Oxalic acid levels in tea culture media as functions of Al and F treatments. (**E**) Tartaric acid levels in tea culture media as functions of Al and F treatments. (**F**) Shikimic acid levels in tea culture media as functions of Al and F treatments. (**G**–**K**) Changes of organic acids, including oxalic acid (**G**), tartaric acid (**H**), citric acid (**I**), malic acid (**J**), and shikimic acid (**K**) in root tissues, as functions of Al and F treatments. Data were from three experiments (*n* = 3) and expressed as means ± SD, and the significant differences between control (0 h time point) and other treatments at 12 h, 24 h, and 48 h time points were analyzed by using the Student’s *t*-test in a two-tailed comparison (* *p* < 0.05 and ** *p* < 0.01). CK—Shigeki Konishi (SK) media containing 0.4 mM Al; Al—2.5 mM Al; F—10 mM F in SK media containing 0.4 mM Al; AlF—2.5 mM Al + 10 mM F.

After pretreatment of the medium samples to remove excessive salts and interfering chemicals, media samples were concentrated and used for the measurement of organic acids in a high-performance liquid chromatography (HPLC) (Figure 1). We detected organic acids in the tea-growth hydroponic media and found oxalic acid, tartaric acid, shikimic acid, and citric acid (Figure 1A,B). The most abundant one was oxalic acid, along with traces of tartaric acid, shikimic acid, and citric acid detected in the tea root-culture media (Figure 1B, Supplemental Figure S1, Table S1). While 0.4 mM Al only induced drastic oxalic acid at 48 h, 2.5 mM Al treatment induced a drastic secretion of oxalic acid from 12 h to 48 h. F treatment initially induced low oxalic acid secretion at 12 h and 24 h, but there was a drastically increased section at 48 h (Figure 1D). A synergistic effect on oxalic acid secretion was observed with Al + F treatment at 12 h, when the content of oxalic acid secretion was at the highest. Furthermore, the secretion of tartaric acid and shikimic acid were slightly induced by 0.4 mM Al at 48 h, but drastically increased by the 2.5 mM Al, 10 mM F, and Al + F treatments (Figure 1E,F). The levels of shikimic acid under Al + F treatment were also synergistically increased at 24 h and 48 h (Figure 1E,F). Al + F treatment induced the highest tartaric acid release at 12 h and 24 h after treatment, significantly higher than both Al and F treatment alone (Figure 1F). With treatment up to 24 h to 48 h, tartaric acid concentrations decreased, but shikimic acid contents in the media increased in the Al + F treatments (Figure 1E,F). Thus, while F treatment displayed toxicity on root organic acid secretion, Al and F treatment together had complex effects on organic acid secretion.

We also analyzed organic acid contents in the roots upon Al and F treatments. In tea roots, oxalic acid, citric acid, and malic acid were the major detected organic acids, while tartaric acid and shikimic acid were the minor components in our assays, with an unidentified peak (Figure 1C, Supplemental Table S1).

In roots grown in regular Shigeki Konishi (SK) nutrient solutions containing 0.4 mM of Al, the organic acids displayed the same trends of increasing during the cultivation. The tartaric acid, citric acid, malic acid and shikimic acid contents increased at 24 h and 48 h in the 0.4 mM Al treatment compared to the control (Figure 1H–K), whereas oxalic acid contents only significantly increased at 48 h treatments (Figure 1G).

Upon 2.5 mM Al treatment, oxalic acid, tartaric acid and malic acid contents were significantly increased in the roots (Figure 1G–J). Citric acid content increased significantly at 12 h and 24 h (Figure 1H,I), whereas shikimic acid contents sharply increased at 48 h under 2.5 mM Al treatment (Figure 1K). In 10 mM F treatment, all organic acid contents were drastically increased. Between 24 h and 48 h, citric acid, malic acid, and shikimic acid content increased, while oxalic acid and tartaric acid content decreased under F treatment (Figure 1G–K). In Al and F combination treatment, most organic acids, such as oxalic acid, tartaric acid and citric acid, reached the highest content at 12 h after treatment (Figure 1G–I). Other organic acids, such as malic acid and shikimic acid, also were increased by Al + F treatment (Figure 1J,K).

### 2.2. Altered Expression of TAC Cycle Genes in Tea Plant Roots under Al and F Stresses

**Figure 2:**
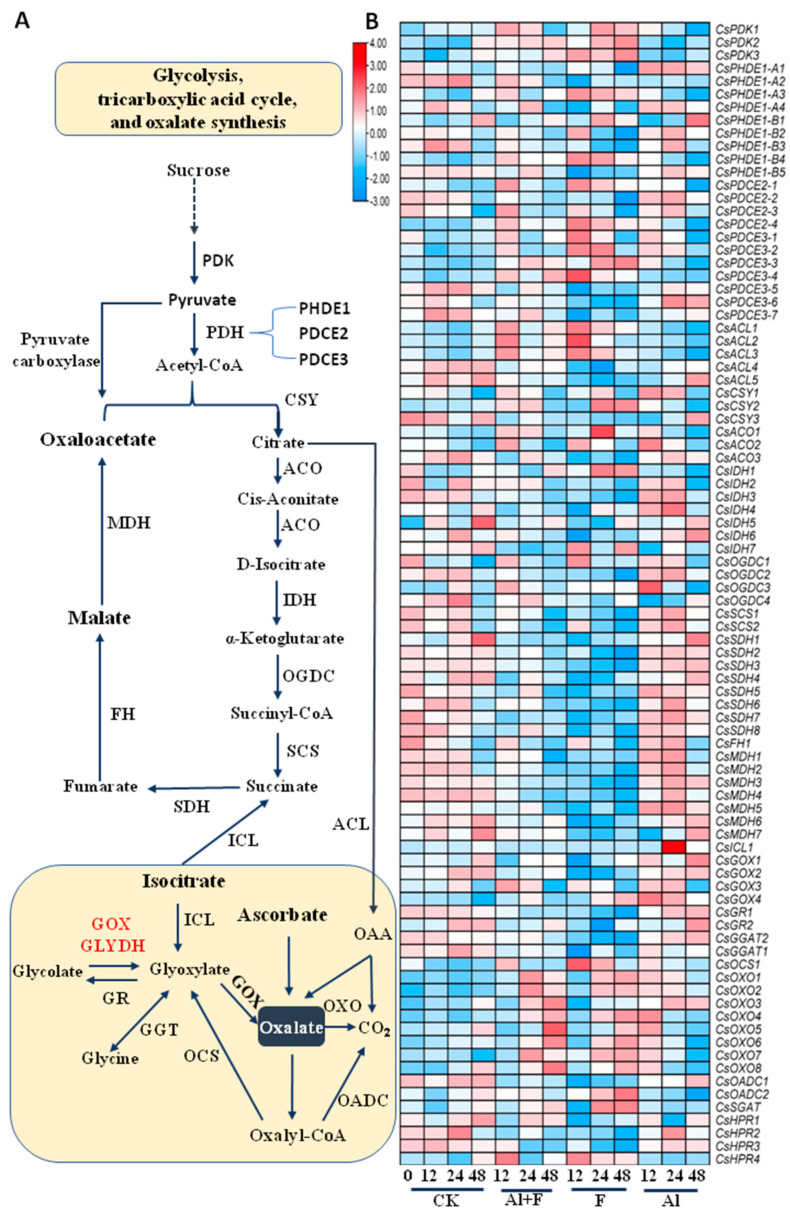
Al and F treatments impacted on tricarboxylic acid cycle and oxalic acid biosynthesis and related gene expression in tea roots. (**A**) The tricarboxylic acid cycle and oxalic acid biosynthesis and involving genes. (**B**) Heatmap analysis of expression patterns of genes involved in catechins biosynthesis in roots at different times after Al and F treatments. Heatmap analysis of gene expression was made with these transcriptome data retrieved from previous transcriptome data, expressed as FPKM (Fragments Per Kilobase of exon model per Million mapped fragments). The Tbtools software was used to structure the heat map. Abbreviations: PDK, pyruvate dehydrogenase kinase; PHDE, pyruvate dehydrogenase; PDCE, dihydrolipoamide acetyltransferase; CSY, citrate synthase; ACO, aconitate; IDH, isocitrate dehydrogenase; OGDC, 2-ketoglutarate dehydrogenase; SCS, succinyl-CoA synthase; SDH, succinate dehydrogenase; FH, fumarase hydratase; MDH, malate dehydrogenase; ACL, ATP-citrate synthase/lyase; ICL, isocitrate lyase; GOX, glycolate oxidase; GR, glyoxylate reductase; GGT, glutamate: glyoxylate aminotransferase; OADC, oxaloacetate decarboxylase; OXO, oxalate oxidase; OCS, oxalyl-CoA reductase.

TAC is a core metabolic system composed of a series of enzymes for the substrate and energy connection of carbohydrate, lipid, and protein biosynthesis and catabolism [37]. Pyruvate is the direct precursor for amino acid and fatty acid biosynthesis, and a plastidial pyruvate dehydrogenase (PDH) and a mitochondrial PDH enzyme complex both catalyze the oxidative decarboxylation of pyruvate to acetyl-CoA, which not only provides the entry point into the TAC cycle, but also supplies acetyl-CoA for acetyl-CoA carboxylase (ACCase) and for malonyl-CoA synthesis, which can be used for fatty acid and flavonoid biosynthesis. Pyruvate dehydrogenase kinase (PDK) regulates the catalytic activity of the mitochondrial pyruvate dehydrogenase complex and links glycolysis with the TAC and ATP generation. Three *CsPDKs* were up-regulated by the F and Al + F treatments (Figure 2A). The pyruvate dehydrogenase complex is composed of multiple copies of three enzymatic components: pyruvate dehydrogenase (PHDE1), dihydrolipoamide acetyltransferase (PDCE2) and lipoamide dehydrogenase (PDCE3). The E1 component of the pyruvate dehydrogenase complex catalyzes the overall conversion of pyruvate to acetyl-CoA and CO_2_ [38]. In plants, exogenous acetate is readily converted into acetyl-coenzyme A (acetyl-CoA) by acetyl-CoA synthase. Plastidic acetyl-CoA synthase is responsible for the majority of the conversion of acetate to acetyl-CoA for use in other metabolic pathways [39] (Figure 2A, Supplemental Figures S2 and S3, Table S2).

Acetyl-CoA is the starting precursor in the TAC cycle (Figure 2A). Acetyl-CoA and oxaloacetate are condensed into citrate by citrate synthetase (CSY). Among the many transcripts from the transcriptome, two highly expressed and increased transcripts—*CsCSY1* and *CsCSY2*—encoding the CSY enzyme were slightly up-regulated by Al stress. *CsCSY2* was also up-regulated by F stress (Figure 2B). Citrate was then converted to *cis*-aconitate, which is an intermediate that can be further converted to isocitrate by the same enzyme aconitase (ACO). Aconitase catalyzes the first dehydration and then rehydration, and its three transcripts were steadily up-regulated by Al and F stresses. Isocitrate is converted to α-ketoglutarate by isocitrate dehydrogenase (IDH), whose four transcripts were mostly up-regulated by Al stress (Figure 2B).

An α-ketoglutarate dehydrogenation complex catalyzes the fourth step in the reaction, in which α-ketoglutarate is converted to succinyl-CoA by α-ketoglutarate dehydrogenase (OGDC), and succinyl-CoA is converted to succinate by succinyl-CoA synthetase (SCS) (Figure 2A). Succinate is converted to fumaric acid by succinate dehydrogenase (SDH), which was encoded by multiple transcripts. Most of them were highly expressed in tea roots (Supplemental Figure S3). The hydration of the C=C double bond that occurs in fumaric acid is catalyzed by fumarate hydratase (FH) to give malate, which is further dehydrogenated to produce oxaloacetate by malate dehydrogenase (MDH). Oxaloacetate further reacts with acetyl-CoA to create citrate to start another round of the TAC cycle. Most of the above genes were up-regulated by Al stress but repressed by F stress, except for *CsIDH1*, *4*, *5*, *7* and *CsOGDC3* being up-regulated by F stress (Figure 2B). Reversibly, citrate could directly feed FA synthesis via citrate hydrolysis to acetyl-CoA and oxaloacetate via ATP citrate lyase (ACL). ACL transcripts were expressed to a greater extent only upon F and F + Al treatment (Figure 2B).

### 2.3. Oxalic Acid Biosynthesis in Tea Plant Roots under Al and F Stresses

In the glyoxylate cycle, isocitrate lyase (ICL) is the first key enzyme of the glyoxylate cycle, and it catalyzes the reversible aldol cleavage of isocitrate to glyoxylate and succinate (Supplemental Figures S2 and S3, Table S2). The glyoxylate cycle is a variation of the tricarboxylic acid cycle and is an anabolic pathway occurring in plants, bacteria, and fungi [40]. The glyoxylate cycle centers on the conversion of acetyl-CoA to succinate for the synthesis of carbohydrates. Oxalate synthesis includes secondary reactions in glyoxylate cycle. Glycolate oxidase (GOX) is considered as an important player in oxalate accumulation in plants (Figure 2A, Supplemental Figures S2 and S3, Table S2). Indeed, several *CsGOX* genes were induced by Al stress. The tea glyoxylate reductase 1 (*CsGR1*) and 2 were slightly enhanced by Al treatment (Figure 2B). The *CsGOX3* and *4* transcripts were increased by F stress. The tea oxalyl-CoA reductase 1 (*CsOCS1*) was also up-regulated by F stress. Interestingly, several oxalate oxidase (OXO) genes were significantly induced by F and Al stresses. Among them, *CsOXO1*, *2* and *3* were up-regulated by the Al + F, F and Al treatments, whereas *CsOXO4* and *5* were drastically up-regulated by Al (Figure 2B, Supplemental Figures S2 and S3, Table S2). Therefore, a whole set of genes involved in oxalate synthesis and metabolism were up-regulated by F stress, supporting that F treatment stimulates oxalate synthesis and secretion into the apoplastic space or culture medium. Activation of oxalate degradation through OXO generates CO_2_ and H_2_O_2_ [40], which is usually regarded as a pathogenesis reaction observed in many plant defense responses to pathogen infections. Oxalate can also be converted to oxalyl CoA by oxalate-CoA ligase (OCL) and then degraded by oxalyl-CoA decarboxylase (OADC) into formyl-CoA and CO_2_ [40]. It would be very interesting to know why Al and F stresses could activate this and trigger the secretion of oxalate into the apoplastic space. This may be related to the modification of the cell wall, such as in pectin modification and oxidative burst. The serine-glyoxylate aminotransferase (*SGAT*), glutamate-glyoxylate aminotransferase (*GGAT*) and hydroxypyruvate reductase (*HPR*) genes were also differently regulated by the Al and F treatments (Figure 2B).

### 2.4. Tartaric Acid Biosynthesis in Tea Plant Roots under Al and F Stresses

**Figure 3:**
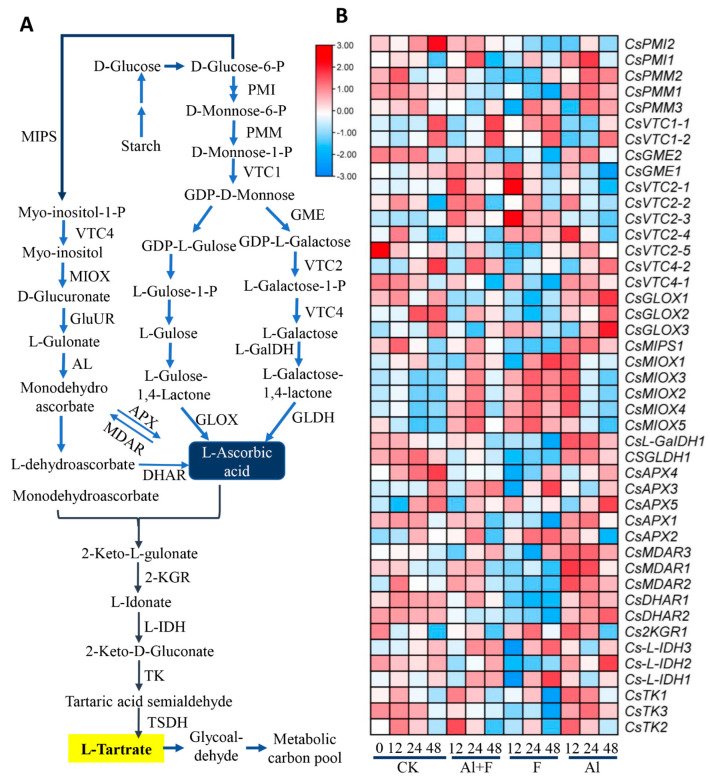
Al and F treatments changed tartaric acid biosynthesis and related gene expression in tea roots. (**A**) The ascorbate-pathway-derived tartaric acid biosynthesis and involving genes. (**B**) Expression patterns of genes involved in tartaric acid biosynthesis in roots at different times after Al and F treatments. Heatmap analysis of gene expression was made with these transcriptome data retrieved from previous transcriptome data, expressed as FPKM. The Tbtools software was used to structure the heat map. Abbreviations: PMI, phosphomannose isomerase; PMM, phosphomannomutase; VTC1, GDP-D-mannose pyrophosphorylase; GME, GDP-D-mannose epimerase; VTC2, GDP-L-galactose phosphorylase; VTC4, L-galactose-1-phosphate phosphatase; L-GalDH, L-galactose dehydrogenase; GLDH, L-galactono-1,4-lactone dehydrogenase; MIPS, myo-inositol-3-phosphate synthase; MIOX, myo-inositol oxidase; GluUR, D-glucuronic acid reductase; AL, aldono-lactonase for L-gulonate; GLOX, L-gulonolactone oxidase; MDAR, monodehydroascorbate reductase; DHAR, dehydroascorbate reductase; APX, ascorbate peroxidase; 2KGR, 2-keto-L-gulonate reductase; L-IDH, L-idonate dehydrogenase; TK, transketolase.

To understand and validate the biosynthesis and secretion of tartaric acid in tea roots, transcriptome data on genes putatively involved in a general pathway towards tartaric acid biosynthesis, which was proposed based on studies on grape and other plants, were collected [41,42,43]. Most studies in plants have shown that tartaric acid biosynthesis in plants is derived from ascorbic acid (Vitamin C), which is also derived from mannose or Myo-inositol [42,44,45]. The majority of the early genes putatively involved in Vitamin C biosynthesis, such as tea phosphomannose isomerase 1 (*CsPMI1*) and 2, phosphomannomutase 1 (*CsPMM1*) and 2, as well as GDP-D-mannose pyrophosphorylase 1 (*CsVTC1*) and GDP-D-mannose epimerase 2 (*CsGME2*) homolog genes were up-regulated by low or higher concentrations of Al (Figure 3A, Supplemental Figures S4 and S5; Table S2). One *CsGME1*, two *CsVTC1*s, GDP-L-galactose phosphorylase 2s (*CsVTC2*s), and one L-galactose-1-phosphate phosphatase (*CsVTC4-1*) were also up-regulated by the F treatment (Figure 3B). The most tea L-gulonolactone oxidase (*CsGLOX*) and myo-inositol-3-phosphate synthase (*CsMIPS*) homolog genes were clearly up-regulated by Al stress only, whereas tea myo-inositol oxidase (*CsMIOX*) homolog genes were drastically up-regulated by both Al and F stresses (Figure 3B). Tea D-glucuronic acid reductases 1 (*CsGluUR1*), 2, and 4 were up-regulated by 0.4 mM Al and F, and only *CsGluUR3* was specifically up-regulated by Al stress. Tea L-galactose dehydrogenase 1 (*CsL-GalDH1*) and L-galactono-1,4-lactone dehydrogenase 1 (*CsGLDH1*) were similarly induced by Al stress, but they were repressed by F treatment. Meanwhile, tea ascorbate peroxidase (*CsAPX*) and 2-keto-L-gulonate reductase (*Cs2KGR*), L-idonate dehydrogenase (*CsL-IDH*) homolog genes were expressed differentially in response to Al and F stresses, most tea monodehydroascorbate reductase (*CsMDAR*) and dehydroascorbate reductase (*CsDHAR*) genes were more significantly induced by 2.5 mM Al stress, and the tea transketolase (*CsTK*) genes were repressed by F stress at 12 and 48 h after treatment (Figure 3B). Thus, these transcriptome data supported the fact that while Al stress clearly induced most Vc and tartaric acid synthesis genes, F could also up-regulate sets of genes for the biosynthesis of Vc and tartaric acid in tea plant roots.

### 2.5. Catechins Biosynthesis Genes Were Differentially Regulated by Al and F Treatments

The shikimic acid pathway uses metabolic precursors, phosphoenolpyruvate from glycolysis and erythrose 4-phosphate from the pentose phosphate cycle to generate 3-deoxy-D-arabino-heptulosonic acid 7-phosphate (DAHP) through a condensation enzyme DAHP synthase (DAHPS), which further generates multiple important metabolites and bioactive molecules. The shikimic acid pathway essentially leads to the biosynthesis of folic acid, salicylic acid and aromatic amino acids (tryptophan, phenylalanine, and tyrosine), as well as other intermediates such as gallic acid, which is glycosylated into β-glucogallin for the biosynthesis of galloylated catechins after condensation reactions with catechins [1].

**Figure 4:**
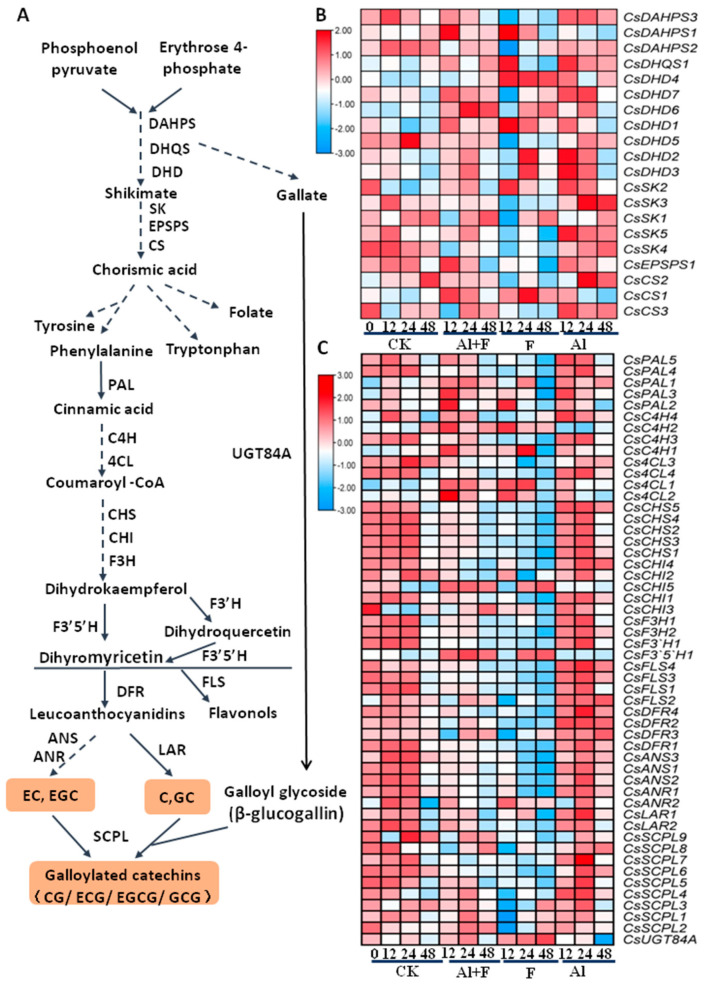
Al and F treatments’ impact on shikimate pathway and catechins biosynthesis genes in tea roots. (**A**) Shikimate pathway and involving genes, as well as phenylpropanoid pathway and involving genes. (**B**) Expression patterns of genes involved in skikimate pathway in roots at different times after Al and F treatments. (**C**) Heatmap analysis of expression patterns of genes involved in catechins biosynthesis in roots at different times after Al and F treatments. Heatmap analysis of gene expression was made with these transcriptome data retrieved from previous transcriptome data, expressed as FPKM. The Tbtools software was used to structure the heat map. Abbreviations: DAHPS, 3-deoxy-D-arabino-heptulosonate 7-phosphate synthase; DHQS, 3-dehydroquinate synthase; DHD, 3-dehydroquinate dehydratase/shikimate dehydrogenase; CS, chorismate synthase; EPSPS, 5-enolpyruvylshikimate 3-phosphate synthase; SK, shikimate kinase; PAL, phenylalanine ammonia-lyase; C4H, cinnamate 4-hydroxylase; 4CL, 4-coumarate CoA ligase; CHS, chalcone synthase; CHI, chalcone isomerase; F3H, flavanone 3-hydroxylase; F3′5′H, flavonoid 3′ 5′-hydroxylase; F3′H, flavonoid 3′–hydroxylase; FLS, flavonol synthase; DFR, dihydroflavonol reductase; ANR, anthocyanidin reductase; ANS, anthocyanin synthase; LAR, leucocyanidin reductase; SCPL, serine carboxypeptidase-like Clade 1A; UGT, UDP-glucose flavonoid 3-O-glucosyl transferase.

As a very essential pathway for both primary and secondary metabolite biosynthesis, the shikimic acid pathway genes were more diverse in their expression patterns in response to Al and F stresses (Figure 4A,B, Supplemental Figures S4 and S5; Table S2). It appeared that Al and Al + F treatment induced a more significant up-regulation of most genes, from some isoforms of tea *CsDAHPS*, 3-dehydroquinate dehydratase (*CsDHD*), 3-dehydroquinate synthase (*CsDHQS*), to downstream shikimate kinase (*CsSK*) and chorismate synthase (*CsCS*) genes (Figure 4B). F treatment caused the repression of more gene isoforms than other treatments, suggesting that F toxicity inhibited gene expression, whereas Al treatment up-regulated the largest number of genes among these treatments.

For genes involving the phenylpropanoid pathway that led to lignin and flavonoid biosynthesis, especially for the biosynthesis of flavonoids in tea plants, Al treatment also resulted in up-regulation to the largest extent, and the most gene isoforms for the pathways from tea phenylalanine ammonia-lyase (*CsPAL*) to serine carboxypeptidase-like Clade 1A (*CsSCPL*) (Figure 4B, Table S2). Particularly, it became more obvious that the majority of the genes involved in flavonoid biosynthesis were up-regulated to the biggest extent in Al-treated roots for 12 and 24 h. Only a small number of genes, including tea chalcone isomerase 5 (*CsCHI5*) and flavonoid 3′ 5′-hydroxylase 1 (*CsF3′5′H1*), and anthocyanidin reductase 2 (*CsANR2*), as well as several genes putatively involved in lignin biosynthesis, such as *CsPAL2* and *3*, cinnamate 4-hydroxylase 1 (*CsC4H1*) and 2, and 4-coumarate CoA ligase 1 (*Cs4CL1*) and 2 were most drastically up-regulated by Al + F and F treatment alone (Figure 4B). Again, 10 mM F treatment repressed more structural genes than other treatments (Figure 4B), indicating the inhibitory effects of F on the biosynthesis of catechins or other flavonoids.

### 2.6. Al and F Stresses Altered the Accumulation of Catechins and Proanthocyanidins in Roots

**Figure 5:**
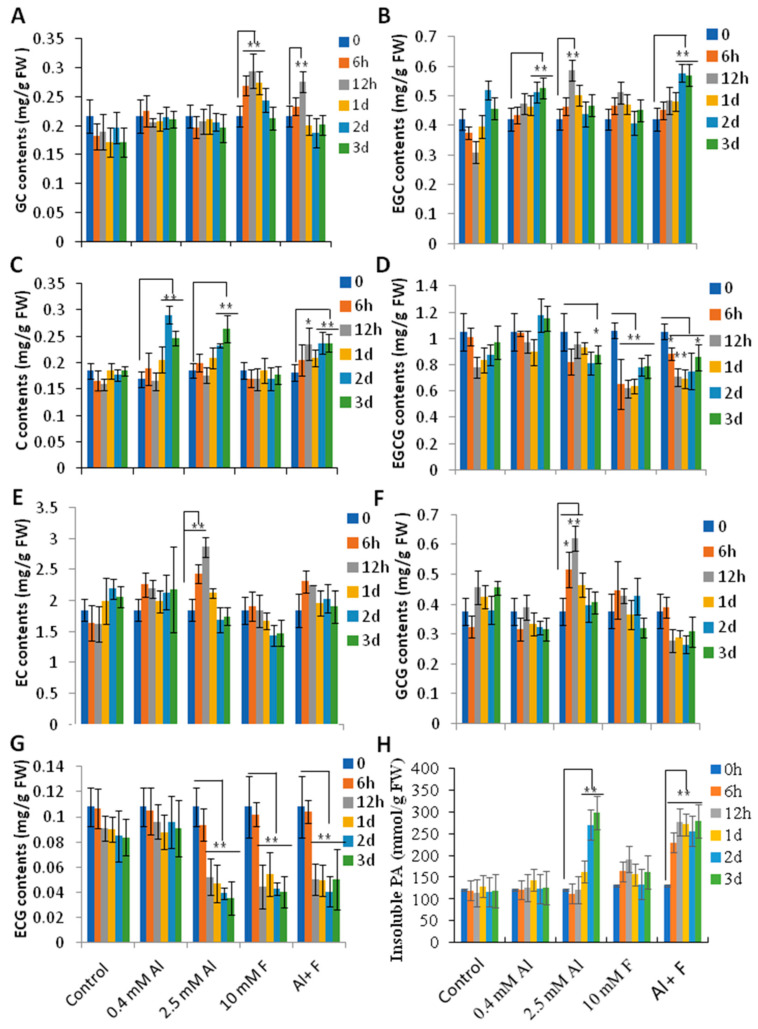
Metabolite profiling of catechins in tea roots under Al and F treatments. (**A**) Changes in GC content in tea roots after Al and F treatments. (**B**) Changes in EGC content in tea roots after Al and F treatments. (**C**) Changes in C content in tea roots after Al and F treatments. (**D**) Changes in EGCG content in tea roots after Al and F treatments. (**E**) Changes in EC content in tea roots after Al and F treatments. (**F**) Changes in GCG content in tea roots after Al and F treatments. (**G**) Changes in ECG content in tea roots after Al and F treatments. (**H**) The changes of insoluble PA contents in roots after Al and F treatments. Data were from three experiments (*n* = 3) and expressed as means ± SD, and the significant differences between control (0 time point) and other treatment times point within groups were analyzed by using Student’s *t*-test in a two-tailed comparison (* *p* < 0.05 and ** *p* < 0.01).

We then assayed the contents of catechins and insoluble proanthocyanidins (also, PAs or condensed tannins) in roots to further understand how Al and F stresses affected their accumulation. Tea roots usually contain large amounts of insoluble PAs as the major form of PAs. Catechins in roots, mainly present in the forms of epicatechin (EC), epigallocatechin gallate (EGCG), gallocatechin gallate (GCG), epigallocatechin (EGC), and catechin (C), and others such as gallocatechin (GC) and epicatechin gallate (ECG), are very minor components. As a minor component of the catechins in tea roots, GC contents were stimulated by F and Al + F treatments (Figure 5A). However, one of the major components in tea roots, EGC, was stimulated by 0.4 mM and 2.5 mM Al, as well as Al + F treatments (Figure 5B). C contents were stimulated by both 0.4 mM and 2.5 mM Al and by Al + F stresses at 2 and 3 days after treatment (Figure 5C). However, EC contents can be generally stimulated to higher levels under 2.5 mM Al, but EGCG contents were reduced by the 2.5 mM Al treatment at Day 3 and by the F treatments (10 mM F alone and Al + F combination) drastically (*p* < 0.05 or 0.01) (Figure 5D,E). GCG contents were also promoted by 2.5 mM Al at 6 and 12 h. ECG contents were, however, mostly repressed by the 2.5 mM Al, F and Al + F treatments (Figure 5G). While these soluble PAs were mostly not drastically changed, insoluble PAs were promoted by 2.5 mM Al and 2.5 mMAl + 10 mM F, indicating that increased stress intensity promoted the accumulation of insoluble PAs in the tea roots (Figure 5H).

### 2.7. Theanine and Caffeine Synthesis Genes Were Differentially Regulated by Al and F Stresses

**Figure 6:**
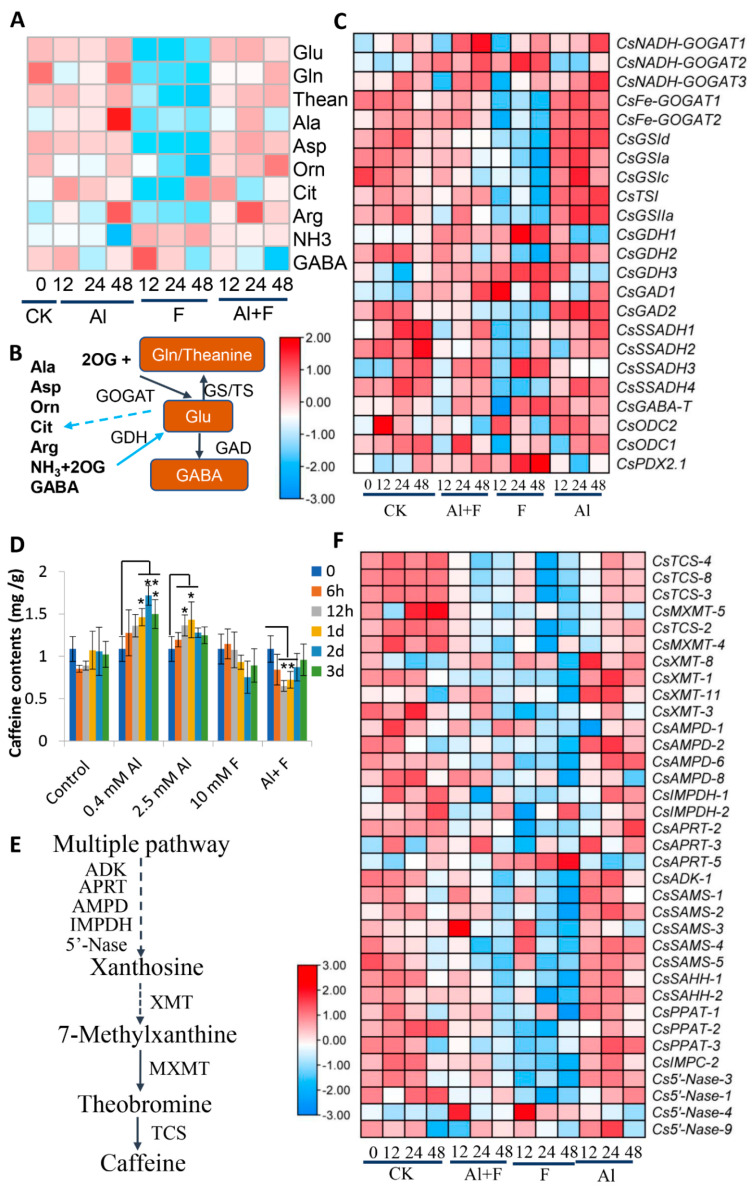
Effects of Al and F stresses on amino acids, theanine, and caffeine accumulation in tea roots. (**A**) Amino acid contents in Al and F treated tea roots for various time. Glutamate (Glu), glutamine (Gln), theanine (Thean), Alanine (Ala), Aspartate (Asp), ornithine (Orn), citrulline (Cit), Arginine (Arg), ammonium (NH_3_), and G-aminobutyric acid (GABA) accumulation in the roots at different times after Al and F treatments. (**B**) Schematic of amino acid metabolic pathways and key genes involved. (**C**) Heatmap analysis of expression patterns of genes involved in amino acid biosynthesis and conversion in tea roots after Al and F treatments. (**D**) Caffeine contents in tea roots after Al and F treatments for various times. (**E**) Caffeine biosynthesis pathway and genes involved in caffeine biosynthesis. (**F**) Heatmap analysis of expression patterns of genes involved in caffeine biosynthesis and conversion in tea roots after Al and F treatments. Heatmap analysis of gene expression was made with these transcriptome data retrieved from previous transcriptome data, expressed as FPKM. The Tbtools software was used to structure the heat map. Data were from three experiments (*n* = 3) and expressed as means ± SD, and the significant differences comparison between o hour and other interval times within group were analyzed by using Student’s *t*-test in a two-tailed comparison (* *p* < 0.05 and ** *p* < 0.01). Abbreviations: GOGAT, glutamate synthase; GS, glutamine synthetase; TS, theanine synthetase; GDH, glutamate dehydrogenase; GAD, glutamate decarboxylase; ODC, ornithine decarboxylase; SSADH, succinic semialdehyde dehydrogenase; GABA-T, GABA transaminase; PDX, L-theanine hydrolase; TCS, tea caffeine synthase; MXMT, theobromine synthase; XMT/7-NMT, 7-methylxanthosine synthase; AMPD, AMP deaminase; IMPDH, inosine monophosphate (IMP) dehydrogenase; APRT, adenine phosphoribosyltransferase; ADK, adenosine kinase; SAMS, S-adenosylmethionine synthases; SAHH, S-adenosylhomocysteine hydrolase; PPAT, amidophosphoribosyltransferase; IMPC, IMP cyclohydrolase; 5′-Nase, 5′-nucleotidase.

We also measured two major N-containing secondary metabolites, caffeine and theanine, in tea roots under Al and F stresses. Organic acid metabolism and amino acid synthesis are fundamentally connected in many pathways as a whole primary metabolic network in most organisms [37,46,47]. For instance, glutamate is formed through the reductive amination of 2-oxoglutarate (2OG) by glutamate dehydrogenase (GDH). The amidation of glutamate to form glutamine by glutamine synthetase (GS) is followed by the reductive transfer of the amide group to 2OG by glutamate synthetase (GOGAT) [1]. The 2.5 mM Al and 2.5 mM Al + 10 mM F could promote the accumulation of amino acids, including glutamine, glutamate, and theanine (Figure 6A, Table S2). However, 10 mM F treatment alone repressed most of these amino acids in the roots (Figure 6A). Correspondingly, two *NADH-GOGAT* genes were up-regulated by Al and Al + F, and transcripts of *Fe-GOGAT* genes were increased by Al treatment, as were *CsGSIa*, *c*, *d*, and tea theanine synthetase I (*CsTSI*), and *CsGSIIa* genes, which were consistent with increased levels of amino acids in the Al treatment (Figure 6A–C). Correspondingly, a *NADH-GOGAT2* and two each of *CsGDH1* and *3* were activated by the F treatment (Figure 6C), and they might have been negatively correlated with theanine and glutamine synthesis; this would be consistent with the negative effects of F on theanine contents in tea shoot tips and the negative impact on tea nutritional quality.

The compound γ-ABA is an important bioactive amino acid and signaling molecule in the plant stress response [48]. Tea glutamate decarboxylase 1 (*CsGAD1*) and *2* were differentially up-regulated by F and Al stresses (Figure 6C), indicating that they differentially contributed to γ-ABA biosynthesis through the degradation of glutamate. Meanwhile, γ-ABA-shunted genes, including tea succinic semialdehyde dehydrogenase 3 (*CsSSADH3*), GABA transaminase (*CsGABA-T*) and L-theanine hydrolase 2.1 (*CsPDX2.1*) and ornithine decarboxylase 2 (*CsODC2*)*,* were activated by F stress and Al treatment (Figure 6C), indicating that they also were attributable to γ-ABA accumulation by the prevention of γ-ABA degradation. Other γ-ABA-shunted genes were up-regulated by Al + F and 0.4 mM Al treatments. Arginine cycle genes, including biosynthesis genes for ornithine, citrulline, arginine, and ammonium (NH_3_), were also altered by Al and F stresses (Figure 6A).

It has been reported that caffeine is found in tea root extrudes under Al stress [25]. Our data also showed that 0.4 mM or 2.5 mM Al drastically up-regulated major caffeine-biosynthesis genes, such as tea caffeine synthase 2 (*CsTCS2*), *3*, *4*, *8*, theobromine synthase 4 (*CsMXMT4*), *5*, 7-methylxanthosine synthase (*CsXMT*), AMP deaminase (*CsAMPD*), inosine monophosphate (IMP) dehydrogenase (*CsIMPDH*), as well as other up-stream biosynthesis genes, including S-adenosylmethionine synthases 1 (*CsSAMS1*), *2*, *S-*adenosylhomocysteine hydrolase (*CsSAHH*), and amidophosphoribosyltransferase (*CsPPAT*) genes (Figure 6E,F). On the other hand, F treatment repressed most of these gene drastically, consistent with the inhibited caffeine accumulation in F- and F + Al-treated tea roots.

### 2.8. Al and F Stresses Affected Tea Secondary Metabolites in Young Tea Leaves

**Figure 7:**
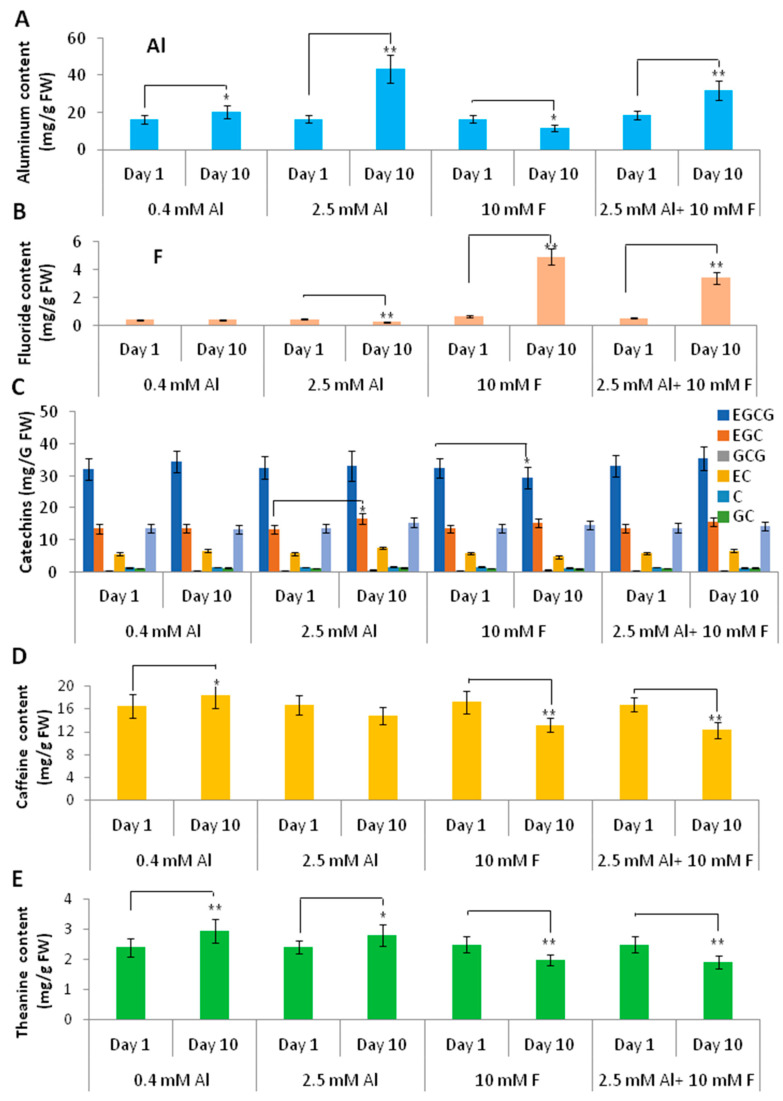
Effects of Al and F stresses on Al/F and tea secondary metabolite accumulation in tea young leaves. (**A**) Al accumulation in the young leaves at day 1 and day 10 after Al and F treatments. (**B**) F accumulation in the young leaves at day 1 and day 10 after Al and F treatments. (**C**) Contents of catechin molecules in the young leaves at day 1 and day 10 after Al and F treatments. (**D**) Caffeine contents in the young leaves at day 1 and day 10 after Al and F treatments. (**E**) Theanine contents in the young leaves at day 1 and day 10 after Al and F treatments. Data were from three experiments (*n* = 3) and expressed as means ± SD, and the significant differences between day 1 and day 10 within groups was analyzed by using Student’s *t*-test in a two-tailed comparison (* *p* < 0.05 and ** *p* < 0.01).

We then examined the Al and F and the tea secondary metabolite contents in young leaves (apical bud and 1st leaf) after 10 days of treatments of Al, F, and Al + F, in comparison with those in the first day of treatments. The tea seedlings grown in 0.4 mM or 2.5 mM Al contained higher Al accumulation in young leaves after 10 days of cultivation compared to the beginning, indicating that Al can quickly accumulate in the young leaves, likely translocated from the roots. However, F treatment alone slightly reduced Al accumulation, whereas F treatment with 2.5 mM Al significantly reduced Al accumulation in young leaves, as compared with 2.5 mM Al treatment alone (Figure 7A), as did F contents in F and Al + F treatment, in which Al treatment reduced F contents in young leaves (Figure 7B). We measured for catechins in the tea young leaves to check the effects of Al and F treatments on tea nutrients. While most of the treatments did not change the contents of the catechin molecules, only the 2.5 mM Al treatment slightly increased the EGC and EC contents at 10 days, but the F treatment inhibited EC and EGCG contents (Figure 7C). For caffeine contents, both the 10 mM F and 2.5 mM Al + 10 mM F treatments reduced the caffeine content in young leaves, perhaps as a result of F accumulation in the young leaves at 10 days after treatments. The 0.4 mM Al tended to increase caffeine levels (Figure 7D). Similarly, theanine contents in 0.4 and 2.5 mM-Al-treated young leaves tended to increase as compared to their control. However, F treatment, either alone or together with Al, reduced the theanine content in the young leaves (Figure 7E).

### 2.9. qRT-PCR Confirmation of Gene Expression Patterns

We also validated the expression patterns for the genes involved in TAC and organic acid and amino acid biosynthesis, and those involved with the catechin, caffeine, and theanine biosynthesis pathways in the tea roots under Al and F treatments. The genes encoding these secondary metabolic enzymes, such as amino acid metabolic genes *CsGDH3* and *CsGSId*; flavonoid metabolic genes *CsCHI4* and *CsDFR4*; tea caffeine synthase *CsTCS4*; and TAC genes *CsCSY1*, *CsGOX1*, *CsOADC1*, *CsL-IDH1*, and *CsTK1*, were examined for their responsive expression patterns to Al and F stresses (Supplemental Figure S6). Most genes displayed altered expression patterns in response to Al and F stresses, which were mostly consistent with transcriptome data (Supplemental Figure S6). The genes encoding for the transporters involved in the secretion of organic acids, including multidrug and toxin extrusion (MATE) and aluminum-activated malate transporter (ALMT), *CsMATE1* and *CsALMT1*, displayed increasing transcript levels from the 2.5 mM Al treatment (Supplemental Figure S6). Although we only observed trace levels of citric acid but no malic acid in the culture media under Al and F stresses, that was probably because they were chelated with Al^3+^ and formed insoluble complexes in the tea root apoplast spaces.

## 3. Discussion

The continuously lowering pH of tea plantation soils is caused by multiple factors, including tea’s characteristic H^+^ secretion mechanism, Al and F stresses, NH_3_ fertilization, and organic acid secretion, or the raising of atmosphere CO_2_/acidic rains. Soil acidification becomes particularly severe after the long-term cultivation of tea plants for 20–30 years, which causes more heavy metal ions to become bioavailable and causes stronger rhizotoxicity in plant roots. The negative impacts of soil acidification and Al and F stresses on tea nutrition value and food safety have been reported. However, the details and mechanisms behind this are not understood. The present study showed that under Al and F stresses, tea roots continuously synthesize and secret oxalic acid, tartaric acid, and minor levels of citric acids, which may chelate extracellular Al for the detoxification of Al/F rhizotoxicity. Catechins, caffeine and theanine biosynthesis were differently stimulated by Al but repressed by F stresses. More importantly, increased Al and F accumulation and generally decreased catechins, caffeine, and theanine contents were observed, indicating that high Al and F concentrations could substantially affect tea nutritional quality and safety.

### 3.1. Tea Roots Had Active TAC Metabolism in Response to Al and F Stresses

Tea roots take up a wide range of inorganic elements or small organic molecules from the soil the aboveground parts of tea plants through vascular tissue transport. Meanwhile roots receive carbohydrates from source leaves through the shoots’ vascular tissue to develop root architectures and support root physiological activities. Understanding the glycolysis, TAC and organic acid metabolism and secretion, as well as the related phenylpropanoid pathway and amino acid metabolism in tea plant roots is critical for the genetic improvement of tea plants for safer teas of higher quality.

The TAC, organic acid and amino acid metabolism pathways provide precursors for the majority of plant primary and secondary metabolism and energy re-generation [49]. As the core metabolic pathway, TAC provides precursors for the biosynthesis of fatty acid for lipids, amino acids for proteins, nucleic acids, polysaccharides for cell walls, the phenylpropanoid pathway towards both lignin and flavonoids, and other plant structures [37].

In plant roots, sources-derived carbohydrates undergo glycolysis to provide energies such as NADPH/NADH, ATP, and metabolic intermediates or precursors for TAC and other metabolism. Under abiotic stress, these pathways improve plant adaptability to survive the adversary environments since defense responses spend energy and cost metabolites [50].

### 3.2. Tea Roots Actively Secreted Organic Acids to Affect Al and F Accumulation

The root organic acids, such as citric acid, malic acid, and oxalic acid, are also involved in plant stress responses by exudation by roots into the rhizosphere and soil, which can improve soil mineral uptake by releasing P ions from the Al-P precipitate and chelating and detoxifying toxic metals such as Al^3+^ [51,52,53]. Both Al and F treatments could induce the secretion of tartaric acid, shikimic acid and oxalic acid from roots. With the F treatment time extended, oxalic acid content decreased in the roots and increased in the media, indicating that oxalic acid may have been exported from the roots into the medium for detoxification (Figure 1D,G). Over time up until 24 h to 48 h, the tartaric acid contents in the media and roots were decreased, indicating that tartaric acid biosynthesis may be suppressed by 2.5 mM Al (Figure 1E,H). Under Al or F treatment, the shikimic acid contents in the roots were similarly changed with those in the media, indicating that shikimic acid was consistently biosynthesized in the root tissues and secreted into the medium (Figure 1F,K). The role of these extracellular organic acids for chelating excessive Al to detoxify Al toxicity is well documented [54,55]; however, their roles in tea roots and the rhizosphere in response to F stress are not clear. Obviously, the secretion and accumulation patterns of these organic acids induced by Al and F stresses are different. It is posited that these extracellular organic acids may inhibit F uptake as competing anions, or may compete with F to form salts with other metal ions such as Al, Fe, Ca, and Mn enriched in acidic soils [7,8,9,20], which is worthy of further investigation.

MATE and ALMT are well-known for the plant root secretion of citric acid and malic acid, respectively, in response to Al or other heavy metal stresses [54,55]. The Al induction of these genes’ expression is regarded as one of the major Al tolerance mechanisms in many plants [51]. Indeed, an overexpression of *TaALMT1* and *HvACCT1* increased the exudation of malate and citrate, respectively, and enhanced aluminum tolerance [56,57]. An overexpression of *MDH* gene enhanced the enzyme activity of MDH, which also increased organic acid synthesis and improved the resistance of alfalfa roots to Al [58]. Our transcriptome data also showed the Al-induced up-regulation of many *CsMATE* and *CsALMT* genes. It has also been reported that tartaric acid can be sequestrated into the vacuole by VvALMT9 in grape berry, indicating that ALMTs could transport both malic acid and tartaric acid [59]. *VvALMT9* homolog genes in the tea genome were also up-regulated at the highest threshold by Al stress; thus, *CsALMT9* homologs could be involved in tartaric acid secretion by tea roots. Although so far, no report on the oxalic acid transporter has been reported, studies showed that two aquaporin transporters, MsPIP2;1 and MsTIP1;1, were positively affected by oxalate secretion from the root tips and Al accumulation in alfalfa root tips [60]. These organic acids also facilitate internal Al detoxification by being transported into root cells for a long-distance transport from roots to shoots through the xylem in the forms of various Al-organic acids complexes [25,54,55]. Thus, they affect eventual F and Al accumulations in tea leaves and thereby affect tea safety. Furthermore, it is clear that under normal conditions, tea roots also actively release organic acids to the root rhizosphere and soils for dissolving mineral elements and for root absorption, or to affect the microbiota in the rhizosphere and the soil for protection roles. Therefore, the release of organic acids by tea roots has multiple roles and is unnecessarily connected to Al or F tolerance.

### 3.3. Al and F Impacted TAC Metabolism and Synthesis of Organic and Amino Acids Differently

The analysis of the transcriptome data for the TAC and oxalate synthesis pathways revealed that Al treatment induced the expression of most metabolic genes, whereas F inhibited the expression of these genes, and Al treatment attenuated the inhibitory effects of F treatment on the expression of many genes, such as *CsCSY1*, *CsMDH3*, which were reported most often in regard to TAC and organic acid metabolism in tea and other plants under Al stress [35,50]. On the other hand, F treatment actually also up-regulated a set of these metabolic genes, which explained why F treatment also induced the root secretion of oxalate and tartaric acid. Interestingly, the genes induced by F treatment were simultaneously repressed by Al stress, including *CsOADC2*, indicating that Al and F stresses targeted different stress signaling pathways, both of which led to the secretion of oxalic and tartaric acids in the roots by activating oxalate synthesis genes. There was an antagonistic effect between Al and F on the TAC and the oxalic and tartaric acid biosynthesis pathway genes. Also, F treatment inhibited the expression of the *CsL-IDH2*, *CsMIPS1* and *CsMDAR1* genes involved in the tartaric acid synthesis pathway, and Al treatment up-regulated these genes; however, an Al + F combined treatment could alleviate the inhibition effects of F treatment (Figure 3).

Reports have shown that high concentrations of F supplies inhibit the secretion of organic acids by tea roots [35]. Meanwhile, organic acids secreted by roots stimulated by Al stress could further solve and release more F, Pi and Al from the rhizosphere soil for tea plants [35]. In the detected organic acids, oxalic but not tartaric acids and citric acids were the major organic acids secreted to the medium, although tea roots produced high levels of citric acid, which could be transported to the stem through vascular tissues.

Our study showed that Al stress up-regulated TAC genes such as *MDH*, *CS* and *GOX* involved in the biosynthesis of organic acids such as oxalic, tartaric, malic, and citric acids in tea roots and promoted the root secretion of oxalic and tartaric acids into the rhizosphere. These findings are somewhat consistent with previous studies [50,61,62]. MDH and CS activities were repressed by F stress (Figure 2 and Figure 4). For most genes involved in vitamin C and tartaric acid biosynthesis, 10 mM F stress and 2.5 mM Al stress seemed to counteract in the up-regulation and down-regulation of their expression. For instance, F treatment repressed *CsMDAR2* and *CsL-IDH2* genes, but Al drastically up-regulated their expression (Figure 3), suggesting that Al released the repression of gene expression by F treatment.

### 3.4. Al and F Stresses Modulated Catechins, Theanine, and Caffeine Synthesis

Tea plants have evolutionarily adapted to acidic soils in tropical regions containing higher Al and F. Tea plants even require low levels of Al or F (<0.4 mM) to gain their optimal root development and growth [63]. Our data also showed that tea root’s most major secondary metabolites, such as catechins, theanine, and caffeine, were synthesized at the highest levels in the 0.4 mM SK media. Without the Al (0 mM Al control) or 2.5 mM Al concentration, there seemed to form a kind of stress on tea plant root growth and normal metabolism. The higher F stress (10 mM F) substantially reduced the accumulation of these secondary metabolites, particularly, theanine production (Figure 5A). This may be one of the reasons that high F causes the reduced tea quality of the young leaves by reducing theanine accumulation in the young leaves, since high F inhibited theanine biosynthesis in the tea roots and likely the translocation to the tea plants’ young leaves.

## 4. Materials and Methods

### 4.1. Plant Materials and Growth Conditions

*Camellia sinensis* (L.) O. Kuntze. *cv.* ‘Shuchazao’ and ‘Chuyeqi’ were used for gene expression analysis and Al and F treatment. Two-year-old tea seedlings were grown in hydroponic SK-nutrient solution in a greenhouse at 20–25 °C, with a light intensity of 1300 μmol m^−2^ s^−1^ and a photoperiod of 18 h per day/6 h per night until new tender roots emerged [15]. The healthy tea seedlings were transferred into fresh hydroponic solutions containing Al and F at different concentrations, e.g., 0, 0.4 mM Al, 2.5 mM Al in Al_2_(SO_4_)_3_, or 10 mM F (F was added to the in hydroponic solutions containing 0.4 mM Al) and 2.5 mM Al + 10 mM F (F was added to the in hydroponic solutions containing 2.5 mM Al) in NaF according previous report [15,64]. The hydroponic solutions were sampled at 0 h, 12 h, 24 h and 48 h for the determination of organic acids and metabolites. The roots and young leaves of these tea seedlings were collected and immediately stored in liquid nitrogen at various time for RNA analysis, ion measurement, and metabolite profiling. For each treatment, five different individual plants were collected for biological duplication under analyses. Details regarding the hydroponic cultivation are shown in the additional files (Supplemental Table S3), as described previously [33].

### 4.2. Determination of OAs Content in High Performance Liquid Phase

In order to understand how tea-plant roots respond to Al and F stresses, the roots of these tea seedlings were collected at various time intervals for organic acid analysis. The media were also collected for the measurement of the cumulative secretion of organic acids. HPLC (Agilent 1100, Santa Clara, CA, USA) was employed for the separation and determination of organic acids. A 5 mL sample was taken, lyophilized, re-dissolved with 0.25 mL hydrochloric acid solution with pH = 1.0, then passed through a 0.45 μm filter membrane, and then tested on the machine. Standard solutions of oxalic acid, citric acid, malic acid, succinic acid, shikimic acid and tartaric acid were prepared with 0.01 mol/L potassium dihydrogen phosphate as solvent. Liquid phase conditions: analytical column—BEH-C18 column (2.1 mm × 50 mm, 1.7 μm). Mobile phase: 0.01 mol/L potassium dihydrogen phosphate solvent. UV detector: G4212-60008 diode array detector. Detection wavelength: 210 nm. Flow rate: 1 mL/min; column temperature: 30℃. Injection volume: 20 μL. The experiment was repeated three times, and an ANOVA test was used for data analysis.

### 4.3. RNA Isolation and Transcriptome Analysis

RNA isolation from tea roots treated with Al and F in various manners were conducted with kits, as described previously [65]. The mRNA quality and purity were assessed using a NanoDrop 2000 spectrophotometer and RNA analyzer (Thermo Scientific, Wilmington, DE, USA) before being used for the construction of libraries using the Illumina TruseqTm RNA Sample Prep Kit method. The RNA sequencing was performed on an Illumina HiSeq2500 platform in triplicate for each sample. The clean data were mapped to the tea plant genome up to 80% by using TopHat2 software (version 2.1.0). Three Fragments per kilobase of transcript sequence per million base pairs sequenced (FPKM) were introduced to qualify the expression levels of the transcripts. The original data had been submitted to the NCBI database with the number PRJNA748249. The number of reads per kilobase per million mapped reads (RPKM) and read counts were calculated using express. Transcriptome data were analyzed and visualized using TBtools (version V1.108).

### 4.4. RNA Isolation and qRT-PCR Analysis

RNA extraction from tea root samples was performed using polysaccharide polyphenol Plant Total RNA Extraction Kit (Tiangen Biotech, Beijing, China). The qRT-PCR analysis was performed using cDNA synthesized using the SweScript RT I First Strand cDNA Synthesis Kit (Takara, Dalian, China) according to the manufacturer’s instruction [66,67]. The house-keeping gene *CsACTIN* (TEA019484) and *CsGAPDH* (TEA003029) were used as an internal reference to normalize target gene expression levels. The SYBR Green qPCR Premix (Universal) was used for qRT-PCR with 20 μL reaction system; ddH_2_O: 8.2 μL, SYBR Green qPCR Premix: 10 μL, forward primers: 0.4 μL, reverse primers: 0.4 μL, and cDNA: 1 μL. Each sample was set up in triplicate, and the relative gene expression was calculated by the 2^−ΔΔCt^ method. SPSS software (version 18) was used for data analysis. The primers are shown in Supplemental Table S4.

### 4.5. Determination of Catechins, Caffeine, and Theanine in Roots and Young Leaves of Tea Plant Seedlings

All amino acids were extracted and quantified using an L-8900 high-speed amino acid analyzer (Hitachi, Ibaraki, Japan) following the protocol [33]. The amino acid standards, such as theanine, glutamine, glutamate, ornithine, and others, were all purchased from Aladdin (Shanghai, China) and Wako Pure Chemical Industries, Ltd. (Osaka, Japan). Catechins and caffeine were profiled with high-performance liquid chromatography (HPLC) according to the method described previously [68,69]. Reference standards EC, EGC, EGCG, C, CG, and GCG were purchased from Sigma-Aldrich (Steinheim, Germany).

### 4.6. Statistical Analysis

The data were from at least three experiments with biological repeats. Statistical analysis was performed using either Student’s two-tailed *t*-test when comparing treatments with controls or multiple comparisons together with the ANOVA multiple range test at the 0.05 probability level. The confidence limits 95 or 99% were defined as the significant between two-tailed data in the Student’s *t*-test.

## 5. Conclusions

In summary, the present study showed that both Al and F stresses altered the TAC, organic and amino acid metabolism, and biosynthesis of secondary metabolites, such as catechins, theanine, and caffeine in tea roots and young leaves differentially. This was because tea plants are grown in the acidic soils of tropic/subtropic regions and tend to acidify tea plantation soils by the secretion of H^+^ under high Al and F concentrations. Our study not only presented the tea root responses to Al/F toxicities via the synthesis and secretion of organic acids to chelate and detoxify excess intercellular or intracellular Al and acidify the rhizosphere, but also the impact on extracellular pH value, inorganic phosphorous and other heavy metals’ solubility and availability to tea roots. Al–F stress-activated TAC and organic acid synthesis or secretion could also affect the biosynthesis of tea secondary metabolites in the tea roots. Moreover, high Al and F stresses negatively affected the accumulation of tea secondary metabolites in the young leaves and thereby their nutrient value. Comparisons of transcriptome data in combination with metabolite profiling did reveal the corresponding metabolic gene expression, which supported and explained the metabolite changes in the tea roots and young leaves under high concentrations of Al and F stress. This study provides new insights into the molecular aspects of Al- and F-stressed tea plants with regards to responsive metabolism changes, tolerance strategy establishment in tea plants, and also their impacts on metabolites in tea leaves that could influence tea nutritional quality and food safety. This study may also facilitate the future genetic improvement of low-Al and -F tea plant varieties.

## Data Availability

Most of the data that support the findings of this study are available in the supplementary materials of this article; the rest will be available upon request from the corresponding author, who will also be responsible for the distribution of the materials integral to the findings presented in this article in accordance with the policy described in the Instructions for Authors: Jian Zhao.

## Supplementary Materials

The supporting information can be downloaded at: https://www.mdpi.com/article/10.3390/ijms24054640/s1.
