# Supplementary material for: Aluminum and Fluoride Stresses Altered Organic Acid and Secondary Metabolism in Tea (Camellia sinensis) Plants: Influences on Plant Tolerance, Tea Quality and Safety
Source: Int J Mol Sci. 2023 Feb 27;24(5):4640. doi: 10.3390/ijms24054640 (PMC10003434; doi:10.3390/ijms24054640)
Supplement: Supplementary file 1 [file ijms-24-04640-s001.zip › Al-F Supplememtly Figure S1-S6.pdf]

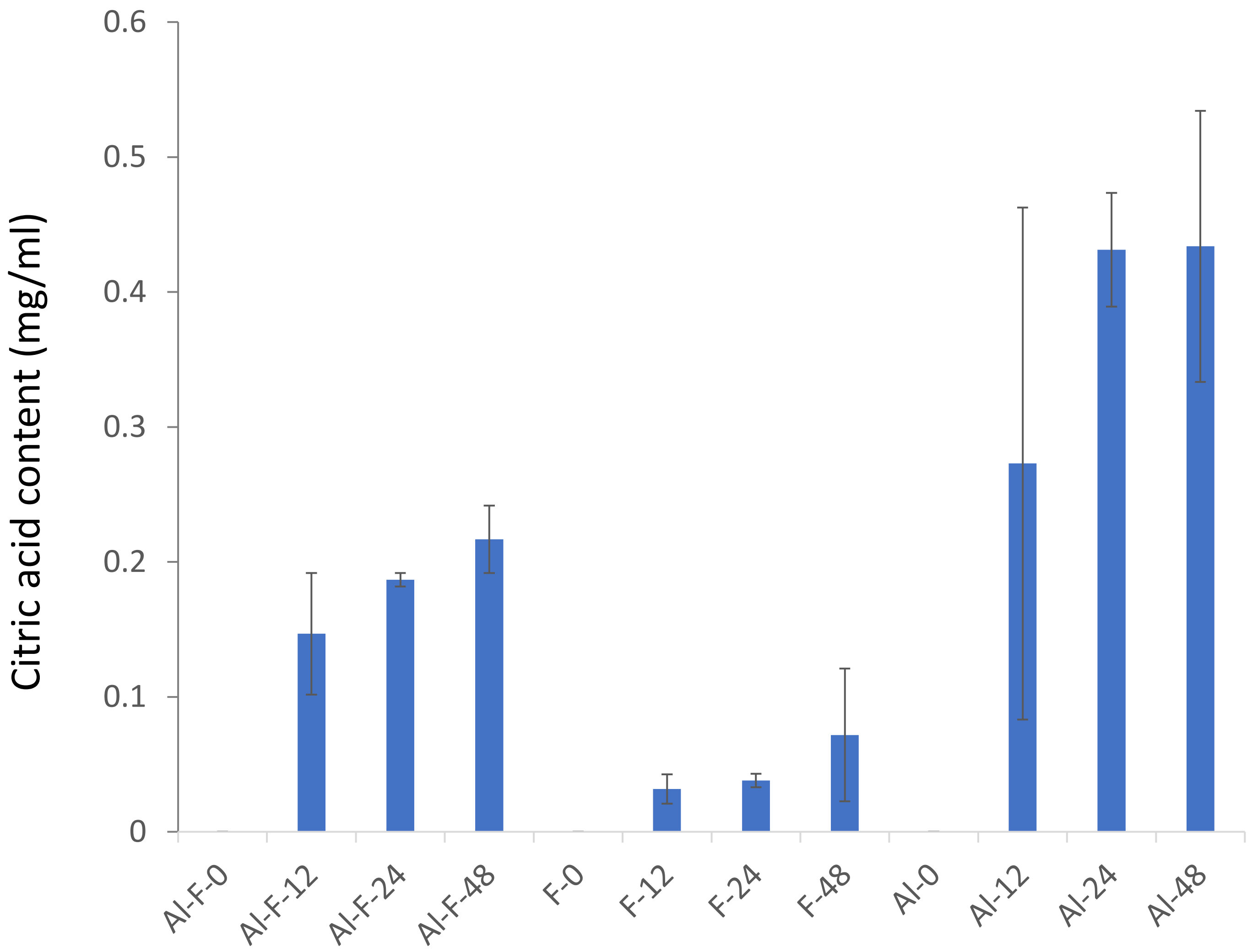

Figure S1. Trace levels of citric acid secretion into tea seedling culture media under Al and F treatments.

Media were collected at different time points after Al and F stress treatments for HPLC analysis. Data are from three independent experiments.

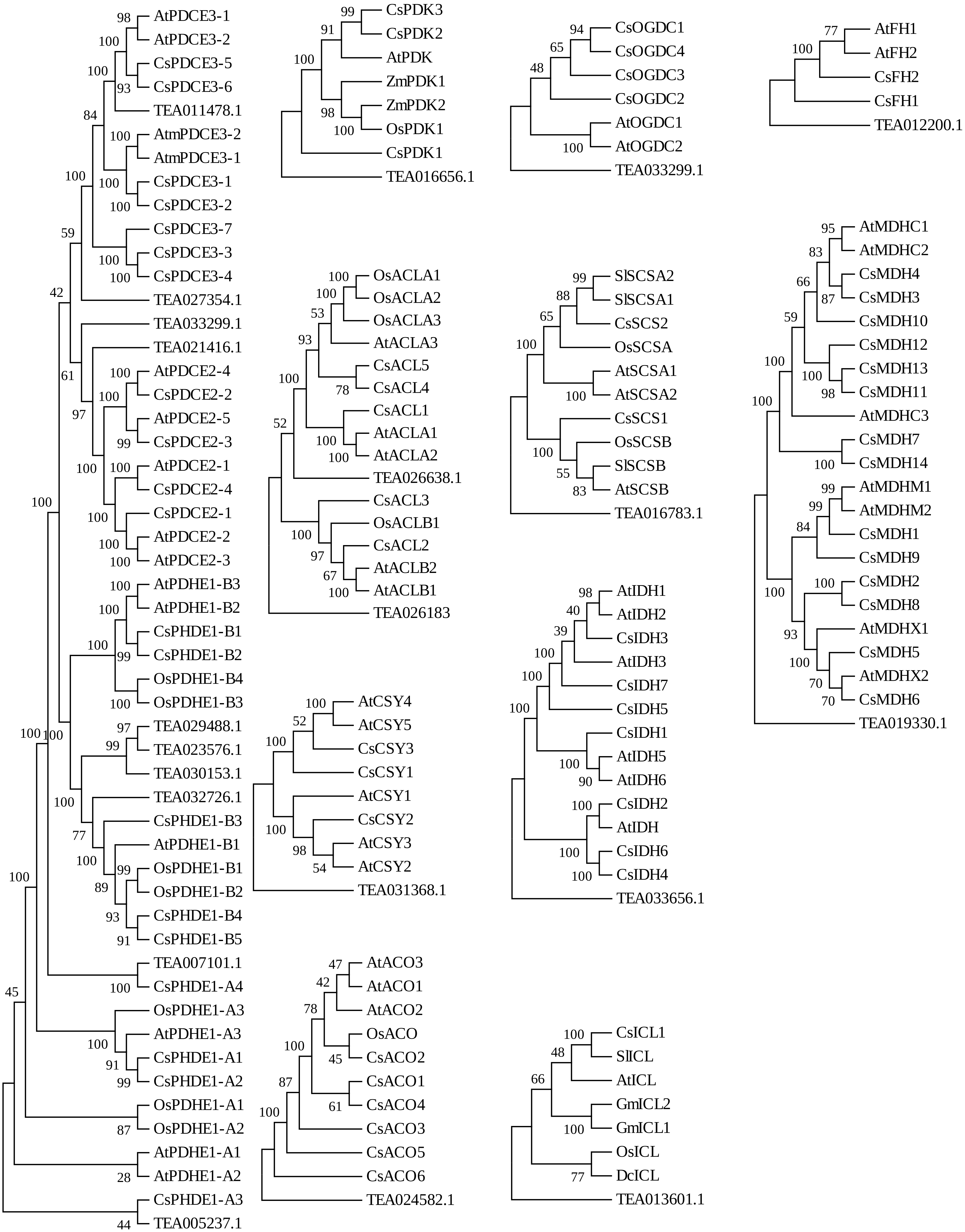

AtPDCE3-1
AtPDCE3-2
CsPDCE3-5
CsPDCE3-6
TEA011478.1
AtmPDCE3-2
AtmPDCE3-1
CsPDCE3-1
CsPDCE3-2
CsPDCE3-7
CsPDCE3-3
CsPDCE3-4
TEA027354.1
TEA033299.1
TEA021416.1
AtPDCE2-4
CsPDCE2-2
AtPDCE2-5
CsPDCE2-3
AtPDCE2-1
CsPDCE2-4
CsPDCE2-1
AtPDCE2-2
AtPDCE2-3
AtPDHE1-B3
AtPDHE1-B2
CsPHDE1-B1
CsPHDE1-B2
OsPDHE1-B4
OsPDHE1-B3
TEA029488.1
TEA023576.1
TEA030153.1
TEA032726.1
CsPHDE1-B3
AtPDHE1-B1
OsPDHE1-B1
OsPDHE1-B2
CsPHDE1-B4
CsPHDE1-B5
TEA007101.1
CsPHDE1-A4
OsPDHE1-A3
AtPDHE1-A3
CsPHDE1-A1
CsPHDE1-A2
OsPDHE1-A1
OsPDHE1-A2
AtPDHE1-A1
AtPDHE1-A2
CsPHDE1-A3
TEA005237.1
CsPDK3
CsPDK2
AtPDK
ZmPDK1
ZmPDK2
OsPDK1
CsPDK1
TEA016656.1
OsACLA1
OsACLA2
OsACLA3
AtACLA3
CsACL5
CsACL4
CsACL1
AtACLA1
AtACLA2
TEA026638.1
CsACL3
OsACLB1
CsACL2
AtACLB2
AtACLB1
TEA026183
AtCSY4
AtCSY5
CsCSY3
CsCSY1
AtCSY1
CsCSY2
AtCSY3
AtCSY2
TEA031368.1
AtACO3
AtACO1
AtACO2
OsACO
CsACO2
CsACO1
CsACO4
CsACO3
CsACO5
CsACO6
TEA024582.1
CsOGDC1
CsOGDC4
CsOGDC3
CsOGDC2
AtOGDC1
AtOGDC2
TEA033299.1
SlSCSA2
SlSCSA1
CsSCS2
OsSCSA
AtSCSA1
AtSCSA2
CsSCS1
OsSCSB
SlSCSB
AtSCSB
TEA016783.1
AtIDH1
AtIDH2
CsIDH3
AtIDH3
CsIDH7
CsIDH5
CsIDH1
AtIDH5
AtIDH6
CsIDH2
AtIDH
CsIDH6
CsIDH4
TEA033656.1
CsICL1
SlICL
AtICL
GmICL2
GmICL1
OsICL
DcICL
TEA013601.1
AtFH1
AtFH2
CsFH2
CsFH1
TEA012200.1
AtMDHC1
AtMDHC2
CsMDH4
CsMDH3
CsMDH10
CsMDH12
CsMDH13
CsMDH11
AtMDHC3
CsMDH7
CsMDH14
AtMDHM1
AtMDHM2
CsMDH1
CsMDH9
CsMDH2
CsMDH8
AtMDHX1
CsMDH5
AtMDHX2
CsMDH6
TEA019330.1

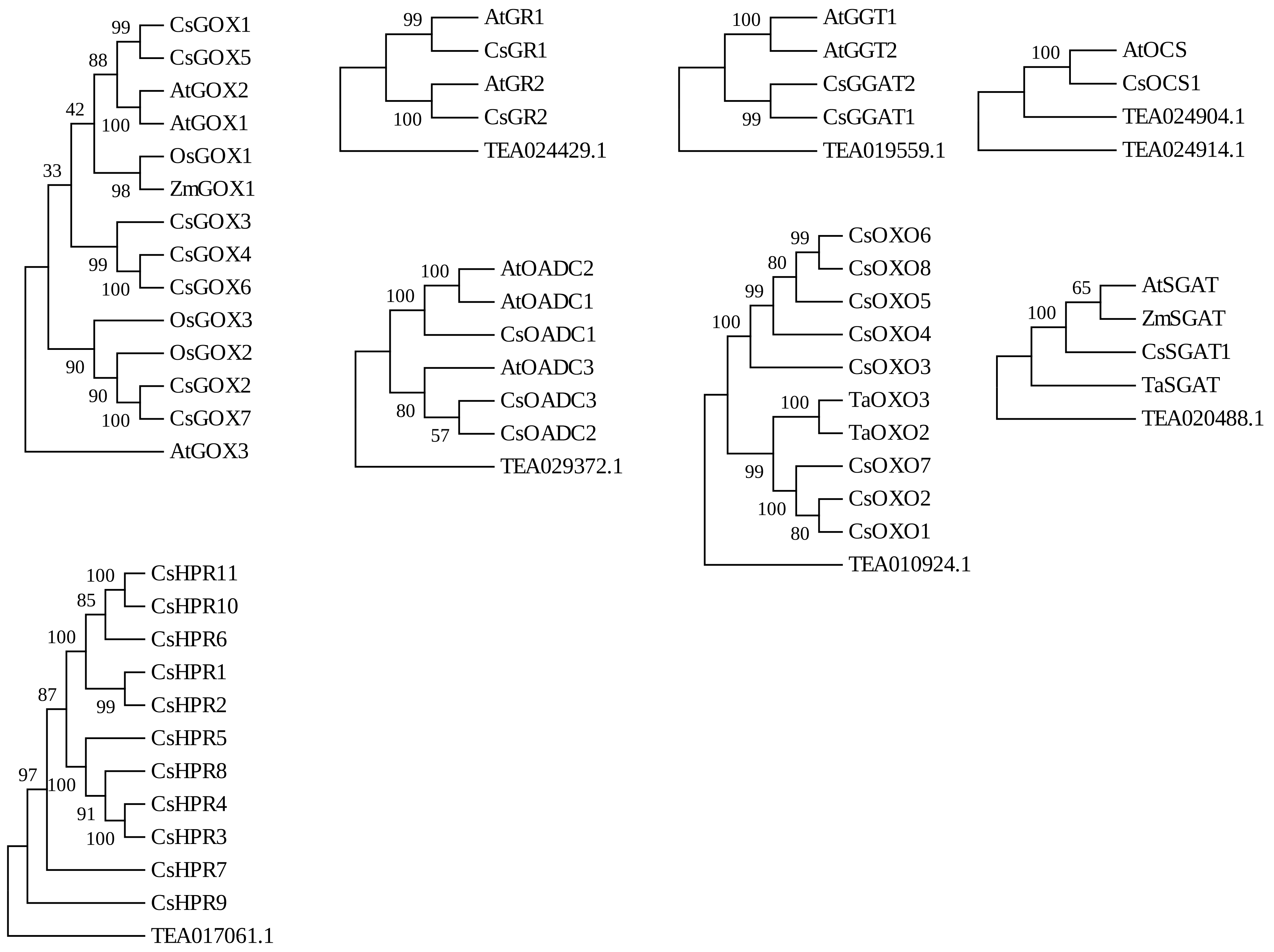

Figure S2. phylogenetic analysis of the key genes involved in oxalate biosynthesis.

PDK, Pyruvate dehydrogenase kinase; PHDE, pyruvate dehydrogenase; PDCE, dihydrolipoamide acetyltransferase; CSY, citrate synthase; IDH, isocitrate dehydrogenase; OGDC, 2-ketoglutarate dehydrogenase; SCS, succinyl-CoA synthase; ACO, aconitate; SDH, Succinate dehydrogenase; FH, fumarase hydratase; MDH, malate dehydrogenase; ACL, ATP-citrate synthase/lyase; ICL, isocitrate lyase; GOX, glycolate oxidase; GR, glyoxylate reductase; GGT, glutamate: glyoxylate aminotransferase ; OADC, oxaloacetate decarboxylase; OXO, oxalate oxidase; OCS, oxalyl-CoA reductase.

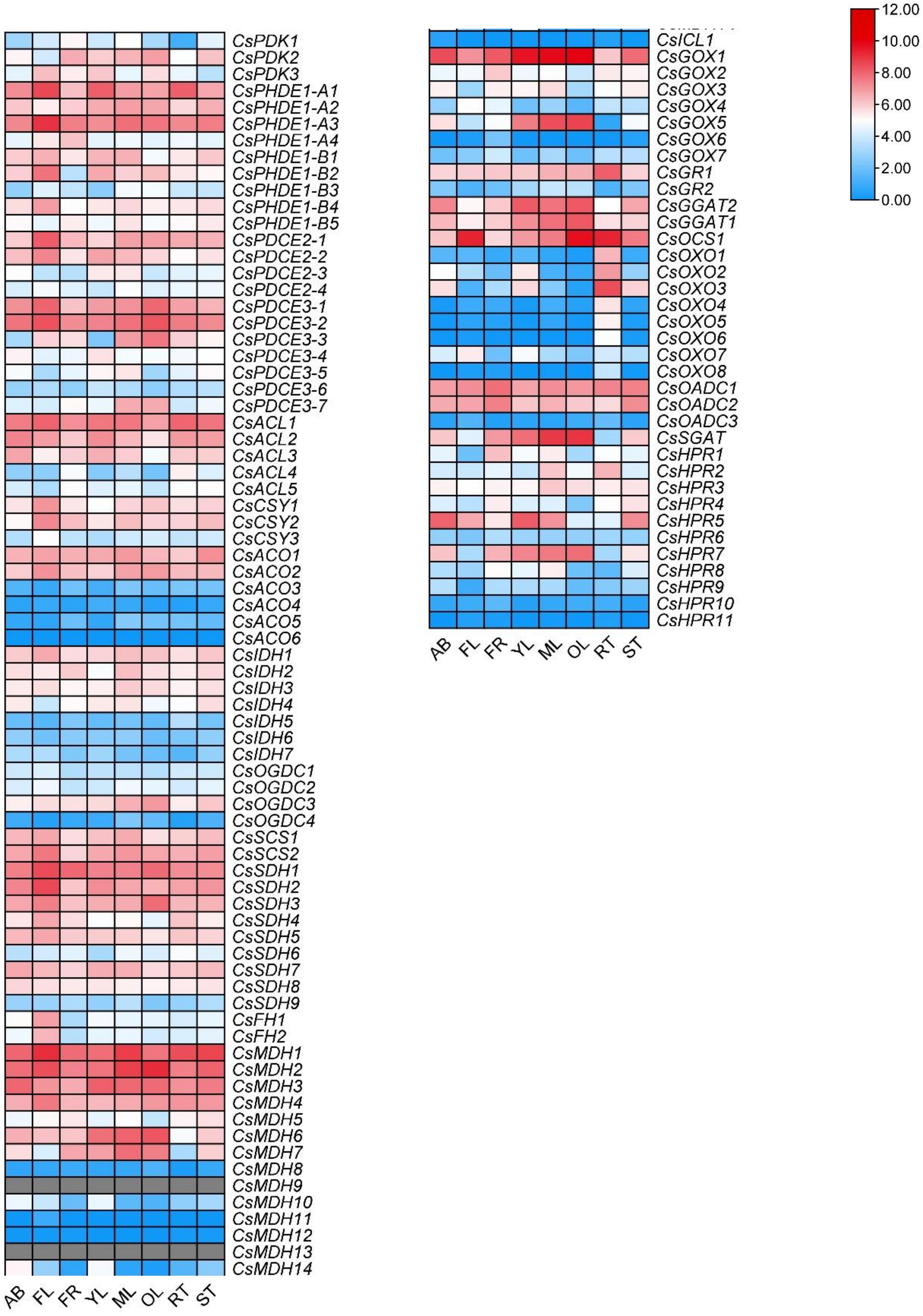

Figure S3. Heatmap analysis of the key genes involved in oxalate biosynthesis in various tissues.

Heatmap analysis of gene expression was made with these transcriptome data retrieved from previous transcriptome data, expressed as FPKM. The Tbtools software was used to structure the heat map.

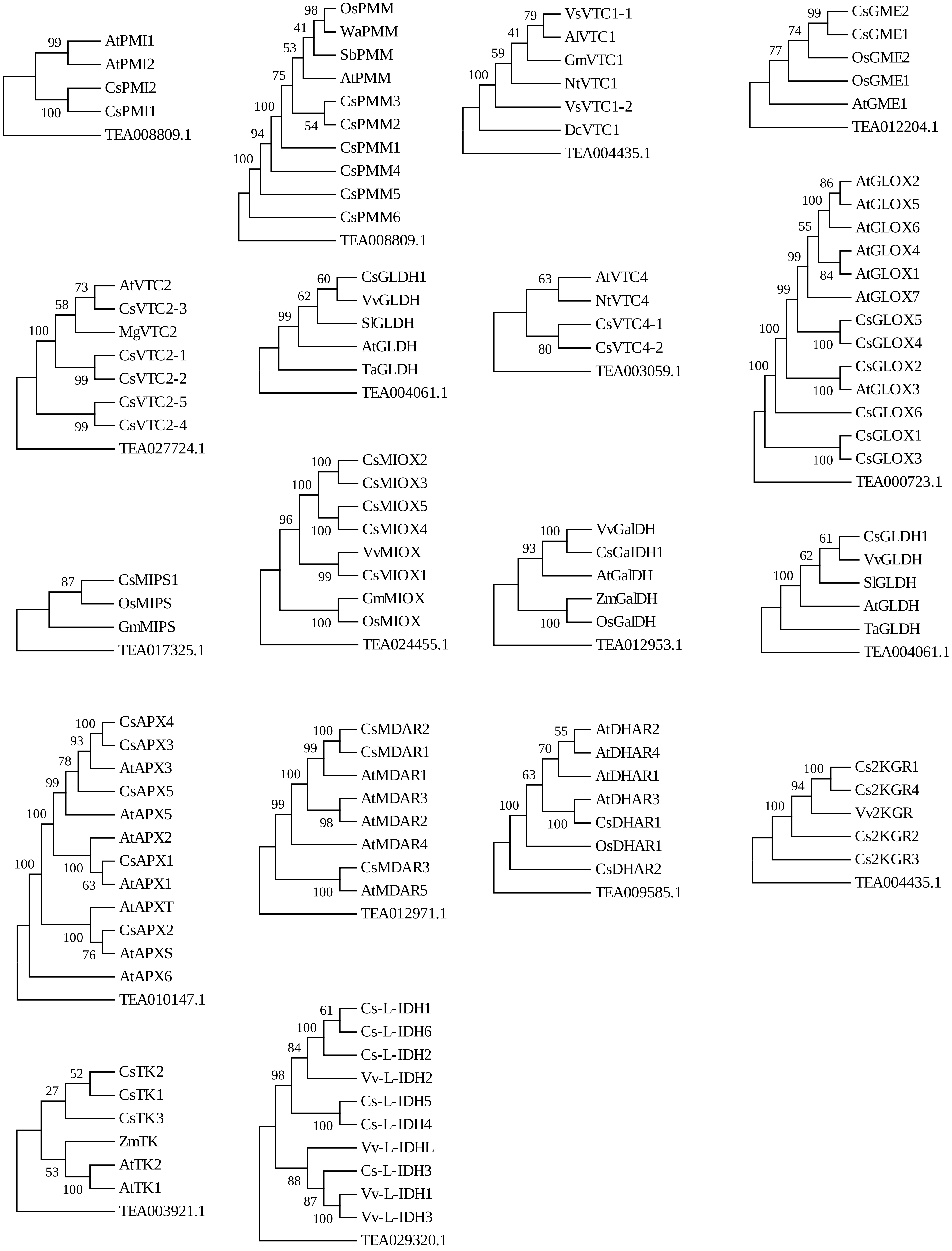

99
AtPMI1
AtPMI2
CsPMI2
100
CsPMI1
TEA008809.1
98
OsPMM
41
WaPMM
53
SbPMM
75
AtPMM
100
CsPMM3
54
CsPMM2
94
CsPMM1
100
CsPMM4
CsPMM5
CsPMM6
TEA008809.1
79
VsVTC1-1
41
AlVTC1
59
GmVTC1
100
NtVTC1
VsVTC1-2
DcVTC1
TEA004435.1
99
CsGME2
74
CsGME1
77
OsGME2
OsGME1
AtGME1
TEA012204.1
86
AtGLOX2
100
AtGLOX5
55
AtGLOX6
99
AtGLOX4
84
AtGLOX1
99
AtGLOX7
100
CsGLOX5
100
CsGLOX4
100
CsGLOX2
100
AtGLOX3
CsGLOX6
CsGLOX1
100
CsGLOX3
TEA000723.1
73
AtVTC2
58
CsVTC2-3
100
MgVTC2
CsVTC2-1
99
CsVTC2-2
CsVTC2-5
99
CsVTC2-4
TEA027724.1
60
CsGLDH1
62
VvGLDH
99
SlGLDH
AtGLDH
TaGLDH
TEA004061.1
63
AtVTC4
NtVTC4
CsVTC4-1
80
CsVTC4-2
TEA003059.1
100
CsMIOX2
100
CsMIOX3
96
CsMIOX5
100
CsMIOX4
VvMIOX
99
CsMIOX1
GmMIOX
100
OsMIOX
TEA024455.1
87
CsMIPS1
OsMIPS
GmMIPS
TEA017325.1
100
VvGalDH
93
CsGalDH1
AtGalDH
ZmGalDH
100
OsGalDH
TEA012953.1
61
CsGLDH1
62
VvGLDH
100
SlGLDH
AtGLDH
TaGLDH
TEA004061.1
100
CsAPX4
93
CsAPX3
78
AtAPX3
99
CsAPX5
100
AtAPX5
100
AtAPX2
100
CsAPX1
63
AtAPX1
AtAPXT
100
CsAPX2
76
AtAPXS
AtAPX6
TEA010147.1
100
CsMDAR2
99
CsMDAR1
100
AtMDAR1
99
AtMDAR3
98
AtMDAR2
AtMDAR4
CsMDAR3
100
AtMDAR5
TEA012971.1
55
AtDHAR2
70
AtDHAR4
63
AtDHAR1
100
AtDHAR3
100
CsDHAR1
OsDHAR1
CsDHAR2
TEA009585.1
100
Cs2KGR1
94
Cs2KGR4
100
Vv2KGR
Cs2KGR2
Cs2KGR3
TEA004435.1
52
CsTK2
27
CsTK1
CsTK3
ZmTK
53
AtTK2
100
AtTK1
TEA003921.1
61
Cs-L-IDH1
100
Cs-L-IDH6
84
Cs-L-IDH2
98
Vv-L-IDH2
Cs-L-IDH5
100
Cs-L-IDH4
Vv-L-IDHL
88
Cs-L-IDH3
87
Vv-L-IDH1
100
Vv-L-IDH3
TEA029320.1

Figure S4. Identification of the key genes involved in tartaric acid biosynthesis in tea plants.

A-R. the key gene’s phylogenetic analysis including PMI, Phosphomannose isomerase; PMM, phosphomannomutase; VTC1, GDP-D-mannose pyrophosphorylase; GME, GDP-D-mannose epimerase; VTC2, GDP-L-galactose phosphorylase; VTC4, L-galactose-1-phosphate phosphatase; L-GalDH, L-galactose dehydrogenase; GLDH, L-galactono-1,4-lactone dehydrogenase; MIPS, myo-inositol-3-phosphate synthase; MIOX, myo-inositol oxidase; GLOX, L-gulonolactone oxidase; MDAR, monodehydroascorbate reductase; DHAR, Dehydroascorbate reductase; APX, ascorbate peroxidase; 2KGR, 2-keto-L-gulonate reductase; L-IDH, L-idonate dehydrogenase; TK, transketolase.

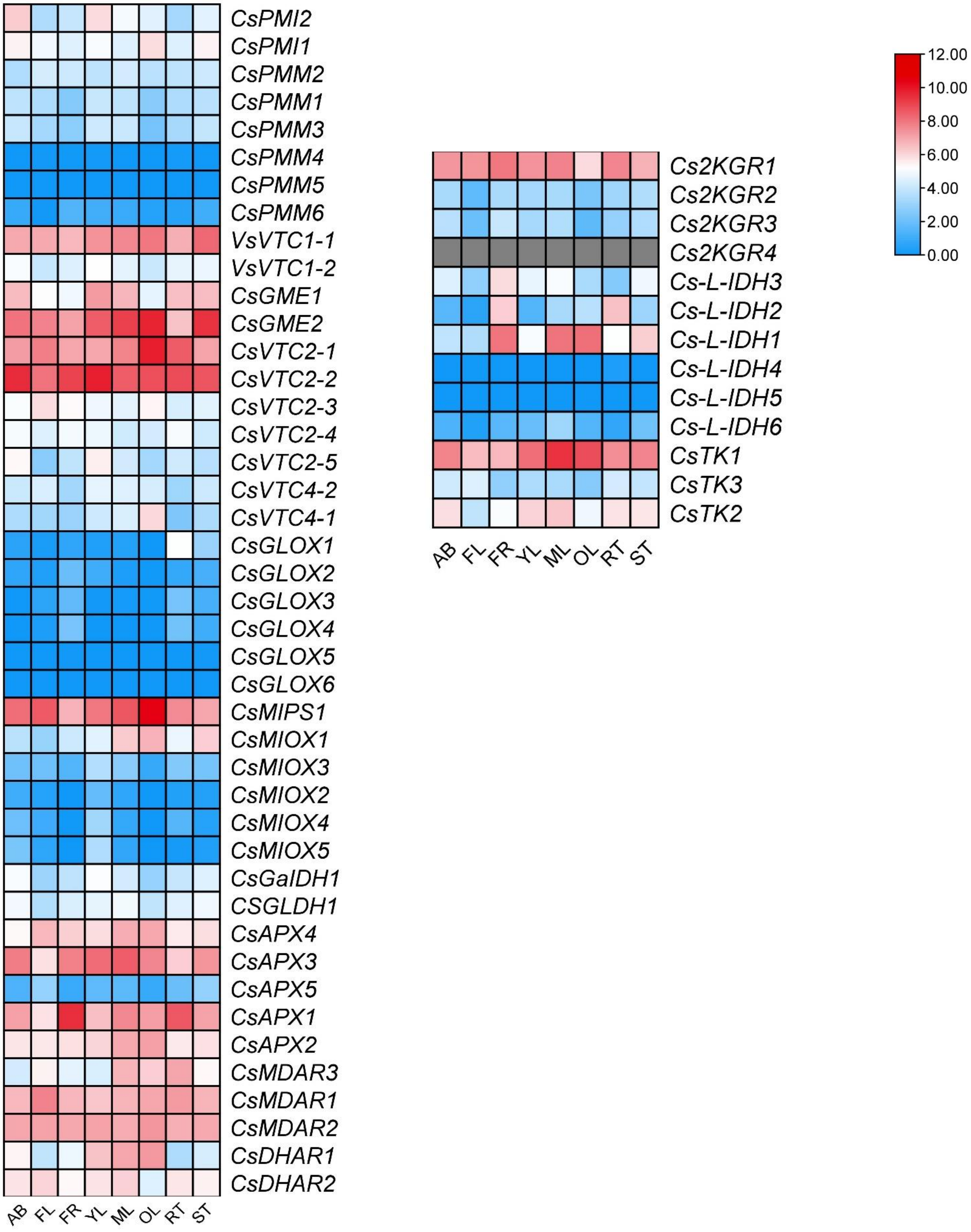

Figure S5. Heatmap analysis of the key genes involved in tartaric acid biosynthesis in various tissues. Heatmap analysis of gene expression was made with these transcriptome data retrieved from previous transcriptome data, expressed as FPKM. The Tbtools software was used to structure the heat map.

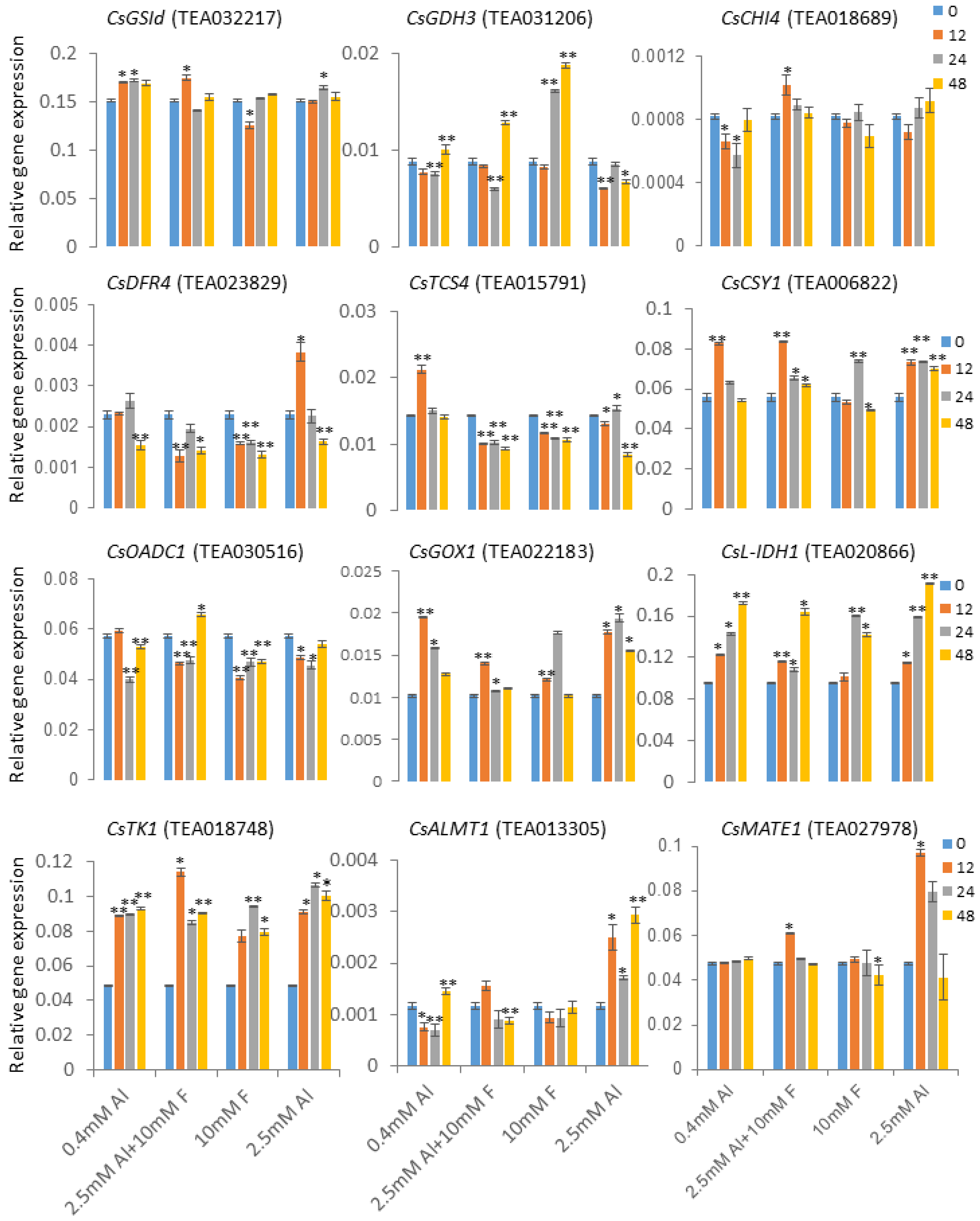

Figure S6. qRT-PCR validation of gene expression patterns in transcriptome.

Tea roots under normal SK medium containing 0.4 mM Al, or Al stress with 2.5 mM Al, F stress medium containing 10 mM F, and combined treatment with 2.5 mM Al and 10 mM NaF (Al+F), were sampled at interval times of treatments for RNA analysis. Both *CsACTIN* and *CsGAPDH* were used as internal references in qRT-PCR analyses. Data were from three experiments (n=3) and expressed as means ±s.d, and the significant differences between comparison was analyzed by using Student's t-test in a two-tailed comparison (*P < 0.05 and **P < 0.01).
